# Effects of Periplasmic Chaperones and Membrane Thickness on BamA-Catalyzed Outer-Membrane Protein Folding

**DOI:** 10.1016/j.jmb.2017.09.008

**Published:** 2017-11-24

**Authors:** Bob Schiffrin, Antonio N. Calabrese, Anna J. Higgins, Julia R. Humes, Alison E. Ashcroft, Antreas C. Kalli, David J. Brockwell, Sheena E. Radford

**Affiliations:** 1 Astbury Centre for Structural Molecular Biology, School of Molecular and Cellular Biology, Faculty of Biological Sciences, University of Leeds, Leeds LS2 9JT, UK; 2 Leeds Institute of Cancer and Pathology, University of Leeds, St. James's University Hospital, Wellcome Trust Brenner Building, Leeds LS9 7TF, UK

**Keywords:** OMP, outer-membrane protein, BAM, β-barrel assembly machinery, OM, outer membrane, tOmpA, transmembrane domain of OmpA, tBamA, transmembrane domain of BamA, POTRA, polypeptide transport-associated, ESI–MS, electrospray ionization–mass spectrometry, MST, microscale thermophoresis, LUV, Large Unilamellar Vesicle, DUPC, 1,2-diundecanoyl-*sn*-glycero-3-phosphocholine, DLPC, 1,2-dilauroyl-*sn*-glycero-3-phosphocholine, DTPC, 1,2-ditridecanoyl-*sn*-glycero-3-phosphocholine, DMPC, 1,2-dimyristoyl-*sn*-glycero-3-phosphocholine, CG-MD, coarse-grained molecular dynamics, OMP biogenesis, Skp and SurA, folding kinetics, native mass spectrometry, coarse-grained molecular dynamics simulations

## Abstract

The biogenesis of outer-membrane proteins (OMPs) in gram-negative bacteria involves delivery by periplasmic chaperones to the β-barrel assembly machinery (BAM), which catalyzes OMP insertion into the outer membrane. Here, we examine the effects of membrane thickness, the *Escherichia coli* periplasmic chaperones Skp and SurA, and BamA, the central subunit of the BAM complex, on the folding kinetics of a model OMP (tOmpA) using fluorescence spectroscopy, native mass spectrometry, and molecular dynamics simulations. We show that prefolded BamA promotes the release of tOmpA from Skp despite the nM affinity of the Skp:tOmpA complex. This activity is located in the BamA β-barrel domain, but is greater when full-length BamA is present, indicating that both the β-barrel and polypeptide transport-associated (POTRA) domains are required for maximal activity. By contrast, SurA is unable to release tOmpA from Skp, providing direct evidence against a sequential chaperone model. By varying lipid acyl chain length in synthetic liposomes we show that BamA has a greater catalytic effect on tOmpA folding in thicker bilayers, suggesting that BAM catalysis involves lowering of the kinetic barrier imposed by the hydrophobic thickness of the membrane. Consistent with this, molecular dynamics simulations reveal that increases in membrane thinning/disorder by the transmembrane domain of BamA is greatest in thicker bilayers. Finally, we demonstrate that cross-linking of the BamA barrel does not affect tOmpA folding kinetics in 1,2-dimyristoyl-*sn*-glycero-3-phosphocholine (DMPC) liposomes, suggesting that lateral gating of the BamA barrel and/or hybrid barrel formation is not required, at least for the assembly of a small 8-stranded OMP *in vitro*.

## Highlights

• Mechanisms of OMP periplasmic transport and folding by BAM are poorly understood.
• BamA catalyzes folding by reducing the kinetic barrier imposed by membrane thickness.
• BamA proteoliposomes promote folding of Skp-bound tOmpA.
• Lateral gating is not required for BamA-catalyzed folding of tOmpA in DMPC bilayers.

## Graphical abstract

**Figure:**
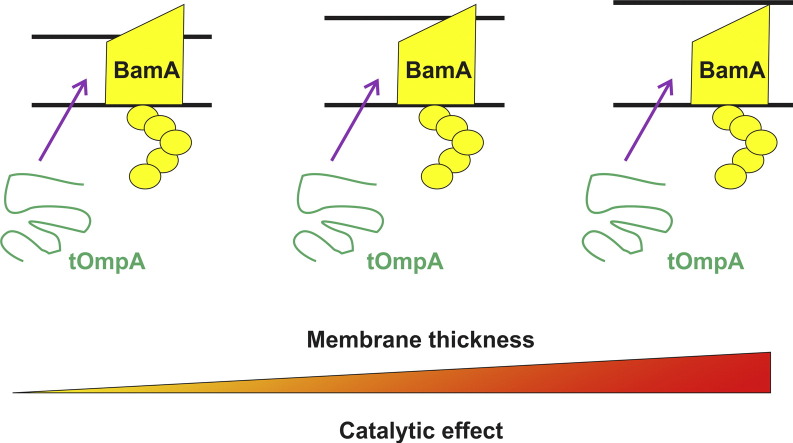

## Introduction

The outer membranes (OMs) of gram-negative bacteria are densely packed with outer-membrane proteins (OMPs), which are involved in a myriad of functions including the uptake of nutrients, release of waste materials, secretion of virulence factors, and resistance to host defence systems [1]. OMPs are synthesized in the cytosol, translocated across the inner membrane, and assisted across the periplasm by chaperones, which prevent their misfolding and aggregation en route to the OM [2]. The final OMP insertion step is mediated by the heteropentameric β-barrel assembly machinery (BAM) complex (BamA-E), by an unknown mechanism [3], [4]. The central BAM subunit BamA consists (in *Escherichia coli*) of a 16-stranded membrane-embedded β-barrel domain preceded by five tandem polypeptide transport-associated (POTRA) domains. BamA is the only BAM complex member for which homologues have been found in all sequenced gram-negative bacterial genomes [5], and BamA-assisted OMP folding has been demonstrated in the absence of other BAM subunits [6], [7], [8]. The molecular details of how OMPs are transported across the periplasm and delivered to BAM, however, remain unresolved [9]. Both OMP folding into the OM and OMP transport across the periplasm occur in the absence of an external energy source, as no ATP is present in the periplasm [10]. The two major OMP chaperones in *E. coli* are SurA and Skp [9], the latter of which is a functional homotrimer [11], [12]. Genetic studies suggest that Skp and SurA operate in parallel pathways [13], [14]. However, while Skp has been cross-linked to the inner membrane in spheroplasts [15], *in vivo* cross-linking of Skp to BAM has not been reported [14]. By contrast, SurA has been cross-linked to BamA *in vivo* [14], [16], supporting the notion that Skp and SurA may cooperate sequentially, with Skp interacting early in the OMP folding pathway, then handing over its substrates to SurA for delivery to BAM [1]. An alternative pathway in which Skp delivers substrates directly to the OM in a BAM-independent mechanism is supported by *in vitro* data showing that Skp can deliver OMPs to negatively charged synthetic bilayers [17], [18] and by recent data which indicate that Skp can fold at least some OMPs into membranes *in vivo* [19]. Recent kinetic simulations of OMP biogenesis, including synthesis, secretion across the inner membrane, chaperone interactions, folding, and degradation, suggest an alternative stochastic model for OMP biogenesis [20]. Modeling of the flux of unfolded OMPs across the periplasm, incorporating kinetic and thermodynamic data from the literature, suggests that, on average, OMPs may make 100s of interactions with SurA, Skp, and other chaperones, which are present in concentrations such that there is always a free chaperone reservoir available for OMP binding [20].

*In vitro* studies of OMP folding in the absence of chaperones/BAM have shown that the physical properties of the membrane can affect OMP folding rates and yields [21]. Shorter lipid acyl chain lengths, increased lipid unsaturation, increased bilayer curvature [22], and promotion of bilayer defects by maintaining the membrane at its transition temperature [23], all increase OMP folding kinetics and folding yield. These data have inspired the hypothesis that physical alteration of membrane properties by BamA may be central to the mechanism of BAM-mediated folding [24]. Indeed, the role of the conserved BamA subunit in overcoming the kinetic barrier to folding imposed by native lipid head groups has been established [6], [7]. A further kinetic barrier to folding is the hydrophobic thickness of the membrane [25], and it has been proposed that one mechanism by which BAM may aid OMP folding *in vivo* is to locally thin the bilayer [7], [26]. Supporting this, the crystal structure of BamA revealed an asymmetric β-barrel, with a narrowed hydrophobic surface on the side of the barrel closest to the β1–β16 seam [26]. A simulation of the β-barrel domain of BamA from *Neisseria gonorrhoeae* in a dimyristoyl phosphatidylethanolamine (*di*_C14:0_PE, DMPE) bilayer exhibited dramatic membrane thinning of 16 Å close to β16 [26], but this was not replicated in recent simulations of full-length *E. coli* BamA in a native OM [27]. To date, therefore, there is no direct experimental evidence for membrane thinning/disordering in the mechanism of action of BAM.

The small number of hydrogen bonds between β1 and β16 observed in the BamA crystal structure suggests a mechanism of BAM-catalyzed OMP folding involving lateral opening of the BamA β-barrel [26], possibly to allow substrates to exit from the BamA lumen, and/or to allow for the formation of a hybrid barrel with an incoming substrate [28]. In support of these hypotheses, molecular dynamics (MD) simulations of the BamA barrel in a DMPE bilayer exhibited lateral opening events [26], [28], and disulfide cross-linking of the BamA barrel was lethal *in vivo* [28] and impaired the folding of OmpT into BAM-containing proteoliposomes *in vitro* [29]. By contrast, it was recently reported that the folding rate of the 8-stranded OmpX was unaffected by cross-linking of the BamA barrel (in the absence of the lipoproteins BamB-E) in liposomes composed of short-chain lipids (*di*_C:10:0_PC containing a mole percentage of 20% *di*_C:10:0_PE) [30]. Recent structures of the BAM complex demonstrated that the BamA barrel can adopt both “lateral open” and “lateral closed” conformations [29], [31], [32], [33], and cross-linking studies established that the BamA barrel can undergo much larger dynamics at the β1–β16 seam than observed in current structures [30]. However, the role of any such gating in BAM-mediated catalysis of the folding of different OMPs remains unclear.

Here, we have investigated the roles of Skp, SurA, BamA, and membrane thickness in OMP assembly using *in vitro* kinetic folding assays combined with native mass spectrometry (MS) and MD simulations. Specifically, we assess the potential handover of OMPs between different chaperones, their delivery to BamA, and the role of membrane thickness and lateral gating in BamA catalysis of folding. We focus on the 171-residue transmembrane domain of OmpA (tOmpA), as an exemplar of a small OMP [7], [8], [23], [34], [35]. The results show that folding of tOmpA from Skp is facilitated by BamA-containing proteoliposomes, despite tight binding to its substrate (nM *K*_d_ [36], [37], [38]). In addition, we show that SurA is unable to release Skp-bound tOmpA, ruling out models suggesting that the chaperones cooperate in an obligate sequential pathway, at least for this substrate. Using kinetic folding assays with lipids of different acyl chain lengths, we also provide direct evidence that BamA catalytic function involves modulation of the lipid bilayer architecture to reduce the kinetic barrier to OMP folding imposed by membrane thickness.

## Results

### BamA catalyzes tOmpA folding in DUPC liposomes

To investigate the mechanism of BamA-catalyzed folding of tOmpA, three constructs were produced: (i) full-length BamA (residues 21–810), (ii) the β-barrel domain of BamA (tBamA) (residues 425–810), and (iii) the soluble POTRA domains of BamA (residues 21–424). Proteoliposomes containing membrane-embedded full-length OmpA were selected as a control, since OmpA has a similar theoretical pI to BamA (~ 5.5 and ~ 5.0, respectively), and also has a periplasmic domain that comprises ~ 50% of the protein mass. Tryptophan fluorescence emission spectra and SDS-PAGE band shift assays indicated that BamA, tBamA, and OmpA could be successfully folded into 1,2-diundecanoyl-*sn*-glycero-3-phosphocholine (DUPC) Large Unilamellar Vesicles (LUVs), following dilution from 8 M urea (Fig. S1a–c), with similar high yields (89.0 ± 2.0%, 88.2 ± 1.2%, and 85.4 ± 1.2%, respectively) (Fig. S2). This enables the effects of the different proteins on tOmpA folding (Fig. S1d) to be directly and quantitatively compared.

Next, the effects of different folding factors on tOmpA folding into DUPC LUVs were analyzed. Pre-incubation of tOmpA with a two-fold molar excess of SurA before delivery to the LUVs had no effect on the observed rate constant (14.9 ± 0.3 x 10^− 3^ s^− 1^ and 12.7 ± 0.5 × 10^− 3^ s^− 1^ for tOmpA folding in the absence or presence of SurA; Fig. S3a, b), as previously reported for PagP [18]. Folding of tOmpA in the presence of the BamA POTRA domains or prefolded tBamA also had little effect on the observed rate constant (15.9 ± 1.0 x 10^− 3^ s^− 1^ and 18.5 ± 2.1 × 10^− 3^ s^− 1^, respectively; Fig. S3c, d). However, in marked contrast with these results, the presence of prefolded full-length BamA increased the observed tOmpA kinetics and resulted in transients requiring an additional exponential term for adequate fitting (Fig. S3e). The observed rate constant for the faster phase (34.6 ± 3.6 × 10^− 3^ s^− 1^; Table S1) is ~ 2-fold faster than that measured for tOmpA alone. Control experiments in which tOmpA was folded into proteoliposomes containing prefolded OmpA indicated that this effect is not simply due the presence of a prefolded OMP in the bilayer (Fig. S3f). The results show that both the membrane-embedded and contiguous soluble POTRA domains of BamA are required for the greatest increase in the BamA-mediated observed tOmpA folding rate.

### Folding of tOmpA from its complex with Skp is dependent on BamA

**Fig. 1:**
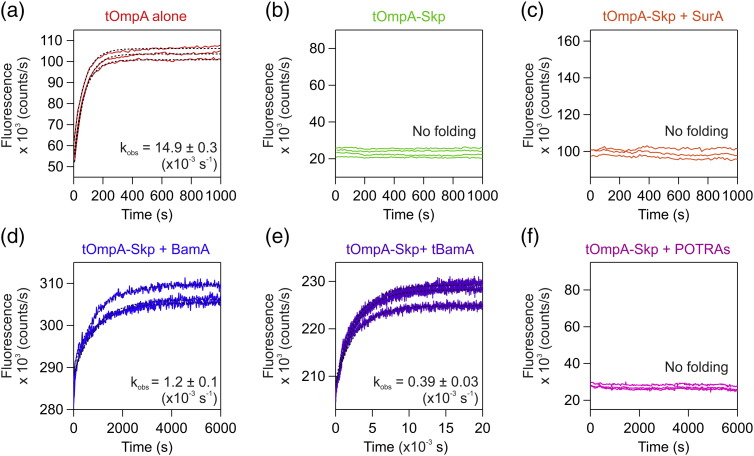
Prefolded BamA promotes folding of tOmpA from its complex with Skp. Kinetic folding traces for (a) tOmpA alone, (b) tOmpA–Skp alone, tOmpA–Skp in the presence of (c) SurA, (d) prefolded BamA (full-length), (e) prefolded tBamA, and (f) BamA POTRA domains. Samples contained 0.4 μM tOmpA, 1.28 mM DUPC, 0.24 M urea, and 50 mM glycine–NaOH (pH 9.5) at 25 °C. A two-fold molar excess (0.8 μM) of Skp, SurA, BamA, tBamA, or BamA POTRA domains was used. A minimum of three transients are shown in each panel. Global fits to a single exponential (Table S2) are indicated by dashed black lines. Note the difference in timescales in different panels.

We have shown previously that tOmpA is prevented from folding into DUPC liposomes (on a 2-h timescale) when pre-incubated with a 2-fold molar excess of Skp [34], where the concentration of Skp is given as trimer equivalents (see Methods). Here, we examined whether addition of SurA or BamA to our assays could result in tOmpA folding from Skp into DUPC membranes. When tOmpA is pre-incubated with Skp, then added to DUPC liposomes in the presence of SurA, no folding was observed as judged by Trp fluorescence, similarly to the results obtained for Skp–tOmpA alone (Fig. 1a–c). Thus, SurA is unable to release Skp-bound tOmpA for folding, consistent with the known lower affinity of SurA for OMPs [39], [40]. Strikingly, and by contrast with the above results, addition of pre-incubated Skp–tOmpA to proteoliposomes containing prefolded BamA resulted in folding, despite the nM affinity of Skp for its OMP substrates [36], [37], [38] (Fig. 1d), with an observed rate constant ~ 12-fold slower than that observed for tOmpA alone (1.2 ± 0.1 x 10^− 3^ s^− 1^ and 14.9 ± 0.3 × 10^− 3^ s^− 1^, respectively; Table S2). No folding was observed in control experiments involving the addition of Skp–tOmpA to proteoliposomes containing prefolded OmpA (Fig. S4), demonstrating that folding of tOmpA from Skp is specifically dependent on the presence of BamA-containing proteoliposomes. These results are consistent with previous SDS-PAGE-based kinetic studies, in which the kinetics of Skp-bound OmpA folding were increased in the presence of prefolded BamA, in LUVs composed of *di*_C12:0_PC (DLPC) with a 20% mole percentage of *di*_C12:0_PE (DLPE) [6].

Next, the ability of the N-terminal POTRA domains or the C-terminal transmembrane domain of BamA to promote folding of tOmpA from Skp were each investigated. tOmpA folding was observed in proteoliposomes containing prefolded tBamA (Fig. 1e), albeit ~ 3-fold more slowly than observed with full-length BamA (Fig. 1d, Table S2). By contrast, tOmpA folding was not observed when Skp–tOmpA was added to a protein construct containing all five POTRA domains in the presence of DUPC liposomes (Fig. 1f). The results show, therefore, that the greatest increase in observed folding of tOmpA from Skp requires the covalent connection between the BamA β-barrel and the POTRA domains.

### Skp-captured tOmpA is not released by SurA

**Fig. 2:**
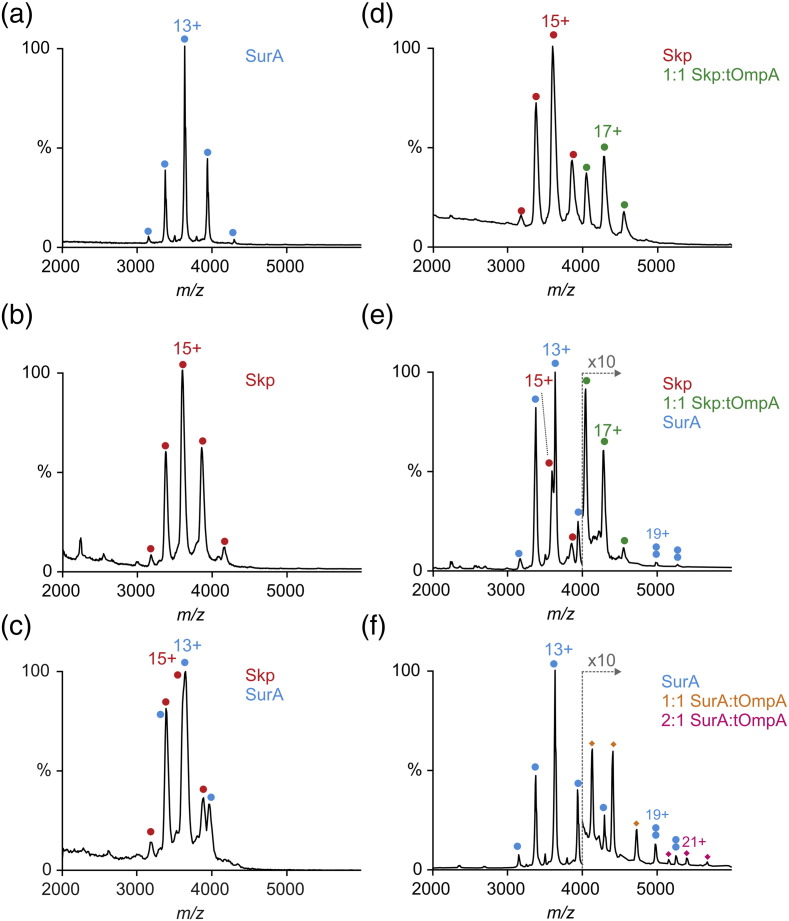
Interactions between SurA, Skp, and tOmpA assessed by native MS. ESI mass spectra of (a) SurA, (b) Skp, (c) pre-incubated Skp and SurA, (d) tOmpA pre-incubated with Skp, (e) pre-formed Skp–tOmpA mixed with SurA, and (f) tOmpA pre-incubated with SurA. Note the expanded intensity scale at > 4000 Da in panels (e) and (f). Peaks corresponding to SurA, Skp, and 1:1 Skp:tOmpA complexes are highlighted in blue, red, and green, respectively. Two blue circles depict SurA dimers. In panel f, peaks corresponding to SurA:tOmpA complexes with stoichiometries of 1:1 and 2:1 are annotated with orange and pink diamonds, respectively. tOmpA was diluted from a denatured state [in 50 mM glycine–NaOH (pH 9.5) and 8 M urea] into Skp or SurA-containing solutions (final tOmpA, Skp, and SurA concentrations all 1 μM, and final urea concentration of 0.2 M) and incubated at room temperature for 5 min before buffer exchange into 200 mM ammonium acetate (pH 10). The most abundant charge state is labeled for each distribution. Observed masses for the complexes are summarized in Table S3.

Next, we used native electrospray ionization (ESI)–MS to investigate more directly whether OMPs bound by Skp can be transferred to, and form a stable complex with, SurA. Gentle ionization conditions were employed to allow non-covalent interactions to be maintained in the gas phase. We sought to observe a binary complex between Skp and SurA, a ternary complex between Skp, SurA and tOmpA, or a transfer of tOmpA from Skp to SurA. ESI mass spectra were acquired for Skp and SurA in isolation (Fig. 2a, b) and pre-mixed in a 1:1 molar ratio (Fig. 2c). A stable Skp–SurA complex was not observed upon mixing, but instead the spectrum obtained was a sum of the spectra obtained for the two chaperones analyzed in isolation, ruling out a stable binary complex. When Skp was pre-mixed with tOmpA at an equimolar ratio, a 1:1 Skp–tOmpA complex was observed in the mass spectrum (Fig. 2d), as shown previously [34]. Addition of SurA to the preformed Skp–tOmpA complex, however, did not result in the formation of a ternary complex between tOmpA, Skp, and SurA, nor did a tOmpA–SurA complex result (Fig. 2e), although tOmpA and SurA form a stable complex in the absence of Skp (Fig. 2f). We further verified that SurA is able to bind tOmpA under the conditions used in kinetic assays using microscale thermophoresis (MST) (Fig. S5). The data were fitted to the Hill equation (Fig. S5), with an apparent binding affinity (*K*_d,app_) of 1.8 ± 0.1 μM. This is in agreement with literature values for SurA binding to a peptide measured by ITC (*K*_d_: 2.3–10.9 μM) [39]. Similarly, competition binding experiments with SurA suggest *K*_I_ values for peptides or full-length OMPs of ~ 0.5–5 μM [40]. However, tighter binding (*K*_d_: 106 ± 84 nM) was observed for SurA binding to the 16-stranded OmpC in FRET-based experiments [38]. The fitted Hill coefficient (1.5 ± 0.1) is suggestive of multivalent cooperative binding, and a poor fit was obtained when the Hill coefficient was held at 1 (Fig. S5). Consistent with this, ESI mass spectra acquired for tOmpA pre-incubated with SurA showed both one and two copies of SurA bound to tOmpA (Fig. 2f), suggesting that SurA may exhibit multivalent OMP binding, as previously demonstrated for Skp [34]. Consistent with the kinetic refolding assays (Fig. 1), the results do not support a mechanism in which SurA acts in a sequential pathway, directly releasing Skp-captured OMPs, but does not rule out transient release of substrate and rebinding to the different chaperones, with the equilibrium in favour of the Skp-bound state [20]. Such behavior is expected based on the known *K*_d_'s of the complexes (μM and nM for tOmpA:SurA and tOmpA:Skp, respectively) and is consistent with recent AFM experiments [41], but counter to hypotheses based on *in vivo* data [1].

### The catalytic effect of BamA is dependent on membrane thickness

**Fig. 3:**
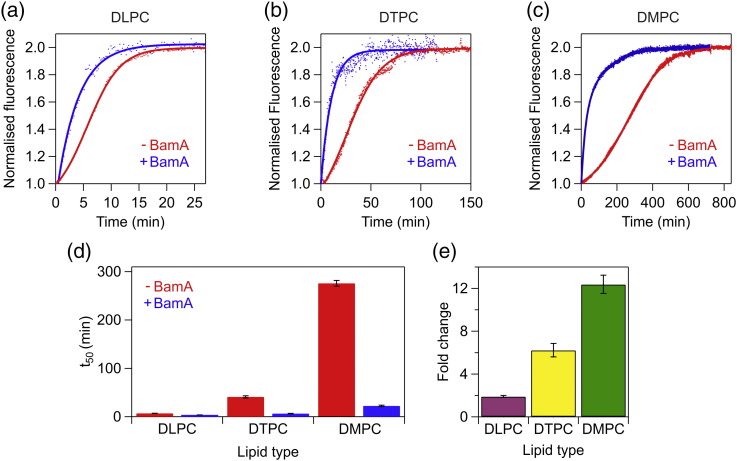
BamA accelerates tOmpA folding more effectively in liposomes with longer acyl chain lengths. Comparison of tOmpA folding in the presence (blue) or absence (red) of BamA in liposomes composed of (a) DLPC, (b) DTPC, or (c) DMPC. Fits to a sigmoidal function (− BamA) or exponential functions (+ BamA) are shown. Note that the timescales of the graphs in (a–c) differ significantly. Raw fluorescence traces are shown in Fig. S7. (d) Comparison of *t*_50_ values for tOmpA folding into DLPC, DTPC, or DMPC liposomes in the presence (blue) or absence (red) of BamA. (e) Fold change in *t*_50_ values between tOmpA folding into DLPC, DTPC, or DMPC liposomes in the presence or absence of BamA. Samples contained 0.4 μM tOmpA, 1.28 mM lipid, 0.24 M urea, and 50 mM glycine–NaOH (pH 9.5) at 30 °C. In BamA-containing samples, a concentration of 0.8 μM BamA was used.

Next, we examined the effect of BamA on the folding kinetics of tOmpA in bilayers created from lipids with different hydrophobic thicknesses: 1,2-dilauroyl-*sn*-glycero-3-phosphocholine (DLPC) (C12, ~ 19.5 Å), 1,2-ditridecanoyl-*sn*-glycero-3-phosphocholine (DTPC) (C13, ~ 21.0 Å), and 1,2-dimyristoyl-*sn*-glycero-3-phosphocholine (DMPC) (C14, ~ 23.0 Å) [42]. If the mismatch between the hydrophobic thickness of the membrane and the BamA barrel domain is important in BamA-facilitated OMP folding, it would be expected that the catalytic effect of BamA would be greatest in thicker membranes. Control experiments showed that the folding yield of BamA in each lipid type is similar (62.7 ± 2.0%, 57.3 ± 2.9%, and 51.7 ± 1.7% for DLPC, DTPC, and DMPC, respectively; Fig. S6a, b and Table S4), as is the secondary structure content of BamA folded into each lipid type (Fig. S6c), allowing direct comparison of the apparent rate of tOmpA folding in the different membrane environments. Folding of tOmpA alone in each of the three lipid types was characterized by a lag phase before a rapid increase in Trp fluorescence, suggestive of the accumulation of one or more folding intermediates (Figs. 3a–c and S7a, c, e) [23], in marked contrast to the behavior observed in DUPC (Fig. 1a). For this reason, observed folding rates in these experiments were compared by measuring the time taken to achieve 50% of the observed fluorescence change (*t*_50_) (see Methods). The results of these experiments showed that the addition of a single methylene group to the acyl chain (~ 2 Å increase in bilayer thickness [42]) has a substantial effect on the observed *t*_50_ of tOmpA; the *t*_50_ is 6 times greater in DTPC compared with DLPC liposomes, while the *t*_50_ into DMPC liposomes is ~ 40 times greater than in DLPC liposomes (Fig. 3d and Table S5). Dramatically, the presence of BamA in DMPC liposomes led to a ~ 12-fold decrease in *t*_50_ (Fig. 3d, e), while ~ 2-fold and ~ 6-fold decreases in *t*_50_ were observed in DLPC and DTPC proteoliposomes containing BamA, respectively (Fig. 3e). Thus, as the hydrophobic thickness of the bilayer increases and is closer to that of the OM [43], the observed folding rate enhancement of tOmpA mediated by BamA increases substantially. To verify that the reduction in *t*_50_ observed in these experiments is specific to BamA, we performed control experiments in which tOmpA was folded into DMPC liposomes that contained prefolded OmpA. Despite the higher folding yield of OmpA in DMPC liposomes under these conditions (88.1 ± 7.0% and 51.7 ± 1.7% for OmpA and BamA, respectively) (Figs. S6a and S7g), the presence of prefolded OmpA resulted in only a 17% decrease in *t*_50_ for tOmpA folding (Fig. S7h, i).

**Fig. 4:**
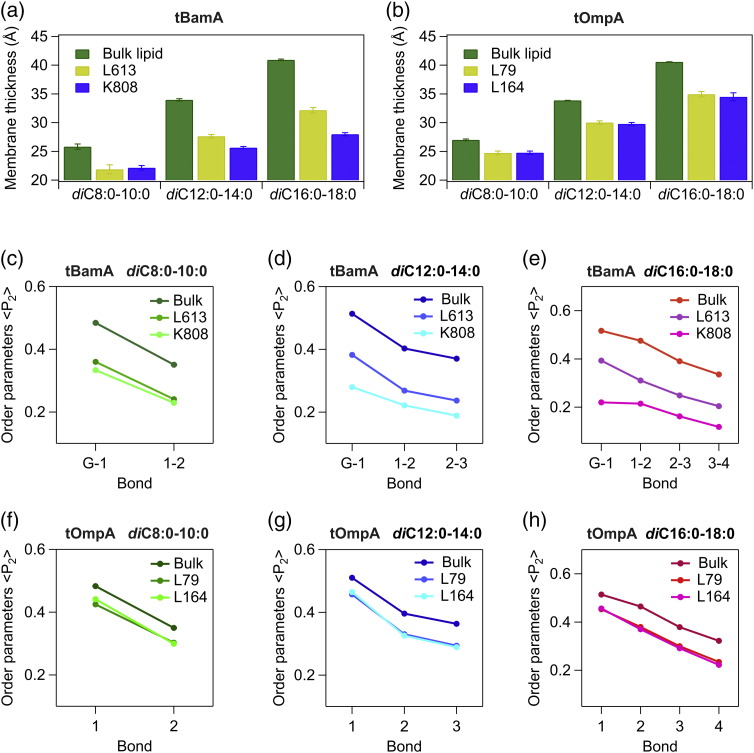
Increases in membrane thinning and lipid disorder mediated by the BamA barrel is dependent on the hydrophobic thickness of the bilayer. (a) Membrane thickness for each of three tBamA-containing simulation systems for lipids within 12 Å of residues L613 (β10) or K808 (β16), or bulk lipid (defined as lipids which are further than 30 Å away from tBamA [45]). Increased membrane thinning is observed close to K808 compared with bulk lipid as lipid chain length increases. (b) Membrane thickness for each of three tOmpA-containing simulation systems for lipids within 12 Å of residues L79 (β4) or L164 (β8), or bulk lipid. (c–e) Bond order parameters for each of the three tBamA-containing simulation systems for lipids within 12 Å of residues L613 or K808, or bulk lipid (see Methods). (f–h) Bond order parameters for each of the three tOmpA-containing simulation systems for lipids within 12 Å of residues L79 or L164, or bulk lipid. Order parameters are shown for the bonds between glycerol particles and first lipid acyl chain particles (G-1), and between consecutive lipid acyl chain particles (1–2, 2–3, and 3–4). Data shown are the mean ± SD from five independent simulations. The duration of each simulation was 3 μs, and data analysis was performed on the final 2.5 μs. Calculated errors in panels c–h are smaller than the size of the symbols.

To investigate the mechanism by which BamA catalyzes OMP folding in thicker bilayers, coarse-grained MD (CG-MD) simulations were performed of tBamA in membranes of different hydrophobic thicknesses, allowing access to μs timescales. Three bilayers were selected containing saturated PC lipids with acyl chains approximately corresponding to *di*_C8:0–10:0_PC, *di*_C10:0–12:0_PC, and *di*_C16:0–18:0_PC (represented by two, three, and four hydrophobic CG particles, respectively; see Methods) [44]. We compared the lipid disorder (using < P_2_ > order parameters) and membrane thickness (using the positions of the particles representing the phosphate group) of the bulk lipid with that of the bilayer in the vicinity of the BamA β1–β16 seam (within 12 Å of residue K808, on β16), and an equivalent residue on the opposite side of the BamA barrel (within 12 Å of residue L613, on β10) (Fig. S8). The results show increased membrane thinning and increased lipid disorder at the tBamA β1–β16 seam compared with bulk lipid (Fig. 4; Tables S6 and S7), consistent with previous atomistic simulations [26]. Importantly, the increases in lipid disorder at the β1–β16 lateral gate are dependent on the thickness of the bilayer, with the greatest effect (2- to 3-fold increase in disorder and ~ 12 Å decrease in membrane thickness compared with bulk lipid) observed in the thickest membranes (*di*_C16:0–18:0_PC) (Fig. 4a, c–e; Tables S6 and S7). Interestingly, the differences in membrane thickness observed between opposite sides of the BamA barrel in the *di*C_12:0–14:0_PC and *di*_C16:0–18:0_PC simulations were small (~ 4 Å) compared with the ~ 16 Å difference observed in previous all-atom simulations at high temperature [26]. Control simulations, in which the effect of the OmpA barrel in each of the membrane environments was simulated, indicated reduced membrane thinning/disordering compared with tBamA (Fig. 4b, f–h; Tables S8 and S9), consistent with the kinetic results (Fig. S7). Importantly, no differences were observed between the lipids in the vicinity of the tOmpA β1-β8 seam and the opposite side of the barrel (Fig. 4b, f–h). Thus, the results support a model in which BamA primes the membrane for OMP insertion at, or near, the β1–β16 seam. The data from both simulations and experiments are consistent with BamA-mediated membrane disruption playing an important role in the catalytic mechanism of BamA and presumably also the BAM complex.

### Lateral opening of the BamA β-barrel is not required for catalysis of tOmpA folding in DMPC liposomes

**Fig. 5:**
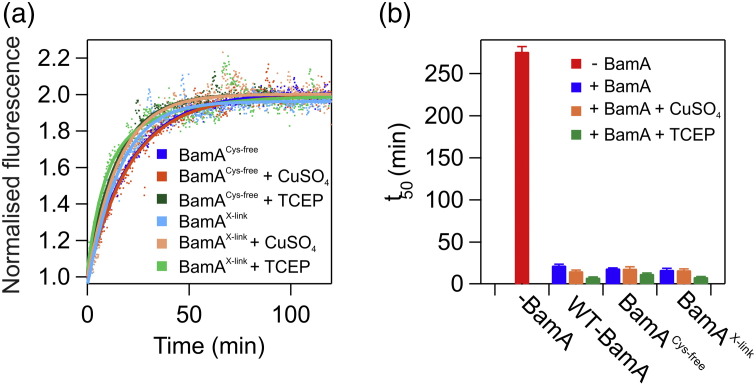
BamA catalysis of tOmpA folding in DMPC liposomes is not dependent on lateral gate opening of the BamA β-barrel. (a) Comparison of fluorescence data for BamA^Cys-free^- or BamA^X-link^-catalyzed folding of tOmpA in oxidizing or reducing conditions. Fits to an exponential function are shown. Raw fluorescence traces are shown in Fig. S9. (b) Comparison of *t*_50_ values for tOmpA folding into DMPC liposomes in the absence (− BamA) or presence of wild-type BamA (WT), BamA^Cys-free^, or BamA^X-link^. Experiments were performed with no additions (blue) or with the addition of 1 mM CuSO_4_ (orange) or 25 mM TCEP (green). Samples contained 0.4 μM tOmpA, 1.28 mM DMPC, 0.24 M urea, and 50 mM glycine–NaOH (pH 9.5) 30 °C. A two-fold molar excess (0.8 μM) of wild-type BamA, BamA^Cys-^^free^, or BamA^X-link^ was used.

Recently, it was shown that the folding rate of OmpX in the presence of prefolded BamA was not affected by cross-linking of the BamA barrel in liposomes composed of *di*_C10:0_PC containing a 20% mole percentage of *di*_C10:0_PE [30]. Here, we used cross-linking to investigate whether opening of the BamA barrel is required for catalysis of tOmpA folding in DMPC liposomes. This lipid was selected as the presence of BamA in DMPC liposomes led to the greatest decrease in observed tOmpA folding *t*_50_ (Fig. 3e), and DMPC bilayers mostly closely match the expected hydrophobic thickness of the OM [43], [46]. Catalysis could involve direct BamA–tOmpA interaction, or be mediated by increased membrane destabilization, which in turn may be caused by opening of the BamA barrel. First, we removed the two native Cys residues in BamA, creating a BamA^C690S/C700S^ mutant (BamA^Cys-free^). In this background, we then introduced mutations designed to cross-link the BamA barrel (I430C on β1 and K808C on β16) creating a BamA^C690S/C700S/I430C/K808C^ mutant (BamA^X-link^). This variant has been shown previously to impair the folding of OmpT *in vitro* by the whole BAM complex [29] and to be lethal *in vivo* under oxidizing conditions [28]. The observed folding *t*_50_ of tOmpA into DMPC liposomes mediated by BamA^Cys-free^, BamA^X-link^, and wild-type BamA was measured in the presence of oxidizing (1 mM CuSO_4_) or reducing (25 mM TCEP) agents (Fig. 5). The results showed that the presence of prefolded BamA^Cys-free^ leads to a similar *t*_50_ for tOmpA folding as wild-type BamA (Fig. 5a), consistent with previous observations *in vitro* [29], [30] and *in vivo* [28], [47]. The observed tOmpA folding *t*_50_ in the presence of BamA^X-link^ was also similar to that in the presence of wild-type BamA and BamA^Cys-free^, both in oxidizing or in reducing conditions (Figs. 5a, b and S9; Table S10), consistent with previous results in C10:0 lipids [30]. Interestingly, the presence of TCEP led to a (~ 2-fold) reduction in *t*_50_ value, compared with in its absence, irrespective of the BamA-variant used (Table S10). The results show that BamA-mediated tOmpA folding catalysis is not dependent on BamA lateral gating and/or BamA:tOmpA hybrid barrel formation, and that opening of the BamA β-barrel in DMPC lipids is not required for modulation of the lipid environment to aid tOmpA insertion and folding, at least under the conditions used.

## Discussion

Recent rapid progress in the understanding of OMP folding and assembly has been made, including the elucidation of the structure of the BAM complex [29], [31], [32], [33], but key questions remain as to the mechanism of BAM-assisted OMP folding and insertion, and the roles, interactions, and mechanisms of the ATP-independent folding factors involved [4], [48], [49]. To address this, an increasing number of folding studies of OMPs have been carried out in the presence of the complete BAM complex [50], [51], [52] and individual folding factors [7], [17], [18], [51]. Here, using kinetic folding assays, we show that BamA is able to promote the folding of Skp-bound OMPs into DUPC membranes despite the nM affinity of Skp for its substrates [36], [37], [38]. It remains to be seen whether this occurs by (1) direct Skp–BamA interaction, (2) interactions of the Skp–tOmpA complex with the BamA-destabilized membrane, or (3) transient exposure of OMPs from the Skp-captured state which interact with either BamA, or a BamA-destabilized membrane region that facilitates OMP release, driven by the tOmpA folding free energy [24], [53]. However, the flux through this pathway is likely to be much lower than through the SurA–BAM pathway given the ~ 1000-fold tighter binding of Skp for its substrates (low nM for Skp [36], [37], [38] and low μM for SurA [39], [40]). This is consistent with recent kinetic simulations showing that when Skp foldase rates are set to be equivalent to those mediated by SurA, the experimentally observed severity of the *∆ surA* phenotype is not reproduced [20]. Interestingly, a recent study in yeast demonstrated that expression of *E. coli* Skp in the mitochondrial intermembrane space assists the assembly of some *E. coli* OMPs (OmpX, PhoE) into the mitochondrial OM, but not others (OmpA) [54], consistent with the view that chaperones and BAM components perform different roles in OMP assembly, depending on substrate [55].

The kinetic and native MS data presented here suggest that *in vivo* SurA is unlikely to release OMPs directly from their Skp-bound state, consistent with competitive affinity assays for the 22-stranded OMP FhuA [41]. The data are consistent, however, with a model proposed for OMP biogenesis in which the populations of chaperone-bound OMPs in the periplasm are controlled by the kinetics of binding and release between OMPs and chaperones, and by their relative concentrations [20]. Direct delivery to the OM or to BAM may be required for Skp-mediated assembly, providing an explanation for the viability of *∆ surA* mutants [14], [56]. Recent MD simulations of Skp in the absence of substrate have revealed that Skp can undergo separation of its three “tentacles” to expand its hydrophobic cavity [34], [57], including a pivoting motion within a conserved region of the coiled-coils of the “tentacles” which may be important for the release, as well as capture, of its clients [57].

The roles of the BamA POTRA domains in OMP assembly also remain unclear. Structural studies indicate that these domains act as a scaffold for BamB-E in the BAM periplasmic ring [29], [31], [32], [33], and *in vivo* cross-linking of SurA to POTRA 1 suggests that the POTRA domains may receive substrates directly from SurA [16]. Here we show that the kinetics of tOmpA folding from its Skp-bound complex are increased in proteoliposomes containing intact BamA compared with the BamA β-barrel alone (Fig. 1d, e). These results are consistent with previous *in vitro* studies, performed in the absence of Skp, in which faster OMP folding was seen in the presence of prefolded BamA compared with prefolded tBamA [6], or a BamA construct lacking POTRA domains 1 to 4 (BamAΔP1–4) [7]. The BamA POTRA domains have also been proposed to chaperone OMP substrates en route to the membrane [58], [59]. Recent evidence from solution NMR demonstrated that the POTRA domains can undergo rigid body motions between POTRAs 2 and 3 [60], and *in vivo* cross-linking data confirmed that flexibility in this region is functionally important [60]. Given that no direct binding of a full-length OMP to the BamA POTRA domains has yet been detected, the incoming OMP may only weakly bind the β-strands of the POTRA domains, allowing OMPs to move toward the BamA barrel and the membrane by processive sliding motions [58], [59]. Recent atomistic MD simulations of full-length BamA in a native OM also showed that its POTRA domains are highly dynamic and that they interact with the membrane independently of tBamA [27]. The observed insertion of the two tryptophan residues in POTRA domain 3 into the membrane [27], which was recapitulated in a recent cryo-EM structure of the BAM complex [29], suggests possible roles for the POTRA domains in modulation of membrane dynamics or in stabilizing the interaction between the periplasmic region of BAM and the membrane during its catalytic cycle, in addition to delivery of OMPs close to the membrane.

Membrane hydrophobic thickness imposes a kinetic barrier to OMP folding [22], [25], and it has been suggested that the decreased thickness of the OM compared with the inner membrane may be one mechanism by which OMPs are sorted to the correct location *in vivo* [25], [37]. The reduced hydrophobic thickness of the BamA barrel in the β1–β16 seam region observed in recent structures of BamA and the intact BAM complex [26], [29], [31], [32], [33], [61] suggests that BamA may assist OMP folding by local perturbations to bilayer thickness [26]. X-ray scattering experiments have shown that the hydrophobic thickness of PC bilayers is linearly dependent on acyl chain length [42], with DMPC bilayers (hydrophobic thickness of ~ 23.0 Å [42]) most closely matching the hydrophobic thickness of the OM as suggested by simulations of native OMs [46] and by the average hydrophobic thickness of a set of 24 OMP structures (23.7 ± 1.3 Å) [43]. The hydrophobic mismatch between the BamA barrel in the β1–β16 seam region and the membrane thus becomes greater as the chain length increases from C12–C13–C14 (with hydrophobic thicknesses of ~ 19.5, ~ 21.0, and ~ 23.0 Å [42]). Here, we have shown that BamA has a greater catalytic effect on tOmpA folding in bilayers containing C14 lipids compared with C13 and C12 acyl chains (Fig. 3), suggesting that hydrophobic mismatch between the BamA barrel and the membrane plays an important role in BamA-mediated tOmpA folding and presumably, therefore, also in the BAM complex. As thicker PC bilayers have lower fluidity at equivalent temperatures, as measured by lipid diffusion rates [62], the data also suggest that BamA may function by locally increasing membrane fluidity. Here, using CG-MD simulations of the BamA barrel in model membranes of increasing hydrophobic thickness, we show that the increased catalytic effect of BamA in thicker bilayers is likely due to increased membrane disruption around the β1–β16 seam (Figs. 3 and S8). The OM contains lipids with varying acyl chain lengths [63], raising the possibility that BAM may actively recruit short-chain lipids to create local areas of thinner membrane to facilitate OMP folding. We have further shown that cross-linking of the BamA barrel does not affect the BamA-mediated catalysis of tOmpA folding in DMPC LUVs, despite the fact that cross-linking of the BamA barrel is lethal *in vivo* [28]. Cross-linking the BamA barrel within a reconstituted *in vitro* BAM complex also impairs the folding of OmpT into proteoliposomes composed of native *E. coli* lipids [29]. Opening of the β1–β16 seam may thus be important for OMP assembly only when BamA is associated with the other BAM subunits or is only required for the assembly of larger OMPs, and/or in more complex lipid membranes than those utilized here.

BamA is an unusual catalyst in that it has two substrates, lipids and proteins [7]. The results presented here establish that BamA-catalyzed OMP folding involves a complex interplay of several factors, including modulation of the bilayer architecture, most likely by local membrane thinning, disruption of lipid packing, and increases in bilayer defects [23], [26]. Our findings also implicate BamA, either directly or indirectly via its effects on the membrane, in facilitating substrate release from Skp. These results do not rule out current models for BAM-mediated OMP assembly involving the formation of substrate barrel-like structures in the periplasm prior to insertion [64], [65], lateral gate opening [26], and/or hybrid barrel formation with incoming substrates [28], [66]. However, we demonstrate that BamA lateral gating is not required for its effect on the lipid bilayer. Further mechanistic insight into BAM conformational changes and interactions with substrates of differing β-barrel sizes during the BAM reaction cycle will be needed to resolve the relative contributions of interactions with OMP folding intermediates and lipid “disruptase” effects in BAM catalysis.

## Methods

### Preparation of liposomes

DUPC (*di*C_11:0_PC), DLPC (*di*C_12:0_PC), DTPC (*di*C_13:0_PC) (DTPC), and DMPC (*di*C_14:0_PC) lipids were obtained from Avanti Polar lipids (Alabaster, AL). Lipids were obtained as a powder, dissolved in a 80:20 chloroform/methanol mixture at 25 mg/mL and stored at − 20 °C until use. Appropriate volumes were transferred to glass test tubes, and an even lipid film was created by drying with a gentle stream of nitrogen while being shaken moderately in a 42 °C water bath. Lipid films were further dried in a vacuum desiccator for > 3 h, followed by resuspension in 50 mM glycine–NaOH at pH 9.5 to a concentration of 40 mM. Resuspended lipids were vortexed briefly and allowed to stand for 30 min. After vortexing again, lipids were subjected to 5 freeze–thaw cycles, with freezing achieved using liquid nitrogen. LUVs (100 nm) were prepared by extruding the lipid suspension ≥ 11 times through a 0.1-μm polycarbonate membrane (Nuclepore, Piscataway, NJ) using a mini-extruder (Avanti Polar Lipids). For DMPC liposomes, the mini-extruder was pre-warmed and the extrusion performed at 37 °C (i.e., above the transition temperate for DMPC; 24 °C) [23]). Liposomes were stored at 4 °C.

### Kinetic folding assays

Kinetic measurements were carried out using a Quantum Master Fluorimeter (Photon Technology International, West Sussex, UK) controlled by FelixGX software v4.3. For each experiment, four separate samples were analyzed in a four-cell changer by a peltier-controlled temperature unit. Tryptophan fluorescence of samples was excited at a wavelength of 295 nm, and fluorescence emission was monitored at 335 nm. The excitation slit widths were set to 0.4–0.6 nm, and the emission slit widths were set to 5 nm. The high emission/excitation slit width ratio was important to minimize photobleaching on the experimental timescale. OMPs were buffer exchanged from 25 mM Tris–HCl and 6 M Gdn–HCl (pH 8.0) into 50 mM glycine–NaOH and 8 M urea (pH 9.5) using Zeba spin desalting columns (Thermo Scientific, UK).

#### Folding experiments in DUPC liposomes

To measure tOmpA folding in the absence of folding factors, folding was initiated by rapid dilution of an 80-μM unfolded tOmpA stock in 8 M urea to a final concentration of 0.4 μM tOmpA and 0.24 M urea in the presence of 1.28 mM DUPC liposomes [a lipid/protein molar ratio (LPR) of 3200:1], in 50 mM glycine–NaOH (pH 9.5) at 25 °C. To measure tOmpA folding in the presence of Skp, SurA, or BamA POTRA domains, tOmpA was first rapidly diluted to a concentration of 2.4 μM in the presence of a two-fold molar excess of each folding factor, in 0.24 M urea, and 50 mM glycine–NaOH (pH 9.5) (no lipids), and incubated for ~ 1 min prior to a further 6-fold dilution in the presence of liposomes. In experiments to monitor tOmpA folding into liposomes containing pre-folded BamA, tBamA, or OmpA, the latter three proteins were first folded for > 1.5 h into liposomes by rapid dilution of each protein from a 100 μM stock in 8 M urea to a final concentration of urea of 0.2 M in 50 mM glycine–NaOH (pH 9.5). Next, unfolded tOmpA was rapidly diluted from an 80 μM stock in 8 M urea to a final concentration of 0.4 μM tOmpA. The final concentrations in these reactions were 0.4 μM tOmpA, 0.8 μM BamA/tBamA/OmpA, 0.24 M urea, 1.28 mM DUPC liposomes, and 50 mM glycine–NaOH (pH 9.5) at 25 °C. The final volume for each sample was 500 μL. In experiments to measure the effects of BamA/tBamA/OmpA on tOmpA–Skp folding, unfolded tOmpA was first rapidly diluted from an 80 μM stock in 8 M urea to a concentration of 2.4 μM in the presence of a two-fold molar excess of Skp in 0.24 M urea and 50 mM glycine–NaOH (pH 9.5) (no lipids), and incubated for ~ 1 min prior to a further 6-fold dilution in the presence of liposomes containing BamA/tBamA/OmpA pre-folded as described, except that the dilution volumes were altered to ensure that the final concentrations in the reactions were identical to those employed in the absence of Skp. The final volume in each sample in experiments to measure the effects of BamA/tBamA/OmpA on tOmpA–Skp folding was 540 μL. At the concentrations utilized here, Skp has been shown to be in a dynamic equilibrium between folded monomer subunits and trimers in the absence of substrate [67]. All Skp concentrations referred to here are trimer equivalents. For each experiment with a particular liposome batch, four samples were measured concurrently. A minimum of three replicates were globally fitted using IgorPro 6.3.4.1 (Wavemetrics, Tigard, OR) to extract rate constant(s), forcing the fits to share the same rate constant(s). Transients were fitted either to a single exponential function:

$$y=A_{1}∙e^{-k_{1}t}+c$$

or to a double exponential function:

$$y=\left(A_{1}∙e^{-k_{1}t}\right)+\left(A_{2}∙e^{-k_{2}t}\right)+c$$

where *k*_1_ and *k*_2_ are rate constants, *A*_1_ and *A*_2_ are their associated amplitudes, and *c* is a constant. Transients were fitted to a double exponential function if a satisfactory fit was not obtained to a single exponential function as judged by inspection of residuals. Experiments were performed for each condition using three separate liposome batches, and reported errors are the standard error of the mean (s.e.m.) of rate constants between liposome batches.

#### Folding experiments in DLPC, DTPC, and DMPC liposomes

tOmpA folding reactions were initiated by manual dilution of an 80 μM unfolded protein stock in 8 M urea to a final concentration of 0.4 μM tOmpA and 0.24 M urea in the presence of 1.28 mM liposomes (an LPR of 3200:1) in 50 mM glycine–NaOH (pH 9.5) at 30 °C. This temperature was chosen to be well above the *Tm* of DMPC liposomes (24 °C), as an increase in tOmpA folding kinetics is observed at temperatures close to the *Tm* [23]. In experiments containing BamA, OmpA, BamA^Cys-^^free^, or BamA^X-link^, the proteins were prefolded overnight by dilution from a 100-μM stock in 8 M urea to a final concentration of 0.8 μM OMP in 0.24 M urea, 1.28 mM DMPC liposomes, and 50 mM glycine–NaOH (pH 9.5) at 30 °C. For reactions containing oxidizing or reducing agents, following overnight folding, 1 mM CuSO_4_ or 25 mM TCEP, respectively, was added to samples and incubated for > 30 min at 30 °C prior to initiation of tOmpA folding. To compare the tOmpA folding data in the presence or absence of BamA, OmpA, or BamA mutants quantitatively, the *t*_50_ value, the time taken to reach 50% of the total fluorescence change on folding was used. A Python script was used to extract *t*_50_ values. For each transient, the minimum fluorescence value was located, and the maximum value was defined by fitting a horizontal baseline to the final section of the data. The *t*_50_ is the time taken to reach a fluorescence value halfway between the minimum fluorescence value and this fitted baseline. At least three separate liposome batches were used for each lipid type, and for each condition (lipid ± BamA), four transients from each lipid batch were used for the *t*_50_ calculation. Errors were calculated as the s.e.m. defined as:

$$s.e.m.=\frac{\sigma }{\sqrt{n}}$$

where *σ* is the SD and *n* is the number of samples. To calculate the error in the fold change in *t*_50_ value with or without the presence of BamA, the s.e.m. was propagated using the following:

$$\mathrm{\delta R}=\left|R\right|∙\sqrt{{\left(\frac{\delta X}{X}\right)}^{2}+{\left(\frac{\delta Y}{Y}\right)}^{2}}$$

where *δR* is the error in the fold change, *| R |* is the fold change value, *X* and *Y* are the mean *t*_50_ values with or without BamA, respectively, and *δX* and *δY* are the s.e.m. values with or without BamA, respectively.

### Fluorescence emission spectra of tOmpA, tBamA, BamA, and OmpA

Fluorescence emission spectra were acquired on the same instrument as the kinetic assays (above). Each spectrum was recorded from 290 to 400 nm in 1 nm increments, using an excitation wavelength of 280 nm. All spectra were acquired at 25 °C, and all samples contained 50 mM glycine–NaOH (pH 9.5) in a sample volume of 500 μL. For unfolded samples, OMPs from a 100 μM stock in 8 M urea were diluted to a final concentration of 0.8 μM in 8 M urea. Folded samples were prepared by dilution of a 100 μM OMP stock to 0.4 μM for tOmpA, or 0.8 μM in the case of tBamA, BamA, and OmpA, in the presence of 1.28 mM DUPC liposomes in 0.24 M urea. The protein concentrations were the same as used in kinetic assays. Samples were incubated at 25 °C for > 1.5 h prior to acquisition of the fluorescence emission spectra.

### Cold semi-native SDS-PAGE OMP band shift assays

BamA, BamA^Cys-free^, BamA^X-link^, tBamA, and OmpA folding efficiency in liposomes was assessed by semi-native SDS-PAGE band shift assays [68]. Tris–tricine gels were made without SDS, and 6 × sample loading buffer was used containing 50 mM Tris–HCl (pH 6.8), 0.1% (w/v) SDS, 0.1% (w/v) bromophenol blue, and 30% (v/v) glycerol. Semi- native SDS-PAGE gels were run in a cold cabinet at 4 °C for ~ 12 h at 14 mA to avoid denaturation of the BamA barrel [68]. Samples contained 0.8 μM OMP, 1.28 mM lipids, 0.24 M urea, and 50 mM glycine–NaOH (pH 9.5) and were folded overnight prior to analysis at either 25 °C for experiments in DUPC liposomes or 30 °C for experiments in DLPC, DTPC, or DMPC liposomes. The fraction of folded OMP was obtained using:

$$\text{Fraction folded}=\frac{\text{Folded band intensity}}{\;\text{Folded}+\text{Unfolded band intensities}}$$

where folded and unfolded band intensities are those in the unboiled lane. We note that, recently, Danoff and Fleming [35] have characterized the appearance of an “elusive state” when monitoring the kinetics of tOmpA folding by SDS-PAGE, as the intensity of the sum of folded and unfolded bands in the unboiled samples decreases over time relative to the boiled band intensity. The authors propose that quantification of the fraction of folded OMP from SDS-PAGE gels be performed by dividing the intensity of the folded band by that of the boiled band. However, in our SDS-PAGE experiments on OmpA, or on BamA and BamA mutants using semi-native SDS-PAGE, following overnight folding we do not observe that the intensity of “F + U” is less than that of the boiled band. Indeed, attempts to use this method yielded fraction folded values of > 100%. In the current work, therefore, we compare fraction folded using the equation above. Note that no kinetic parameters are derived from these gels. Densitometry was performed using ImageJ.

### Mass spectrometry

Samples of SurA and Skp were prepared for MS by buffer exchanging into 200 mM ammonium acetate at pH 10 using Zeba spin desalting columns (Thermo Scientific) immediately prior to analysis. Skp–tOmpA or SurA–tOmpA complexes were prepared by rapid dilution of denatured tOmpA [400 μM in 8 M urea, 50 mM glycine–NaOH (pH 9.5)] to a final concentration of 1 μM into a solution of Skp or SurA [1 μM in 50 mM glycine–NaOH (pH 9.5)]. The samples were subsequently buffer exchanged into 200 mM ammonium acetate at pH 10 using Zeba spin desalting columns.

Spectra were acquired using a Synapt HDMS mass spectrometer (Waters Corporation, UK) by means of nano-ESI using in-house prepared platinum/gold-plated borosilicate capillaries. Typical instrument parameters include the following: capillary voltage, 1.2 kV; cone voltage, 120 V; trap collision voltage, 10 V; transfer collision voltage, 10 V; trap DC bias, 20 V; and backing pressure, 4.5 mBar. Data were processed using MassLynx v4.1 and UniDec [69].

### MST binding experiments

#### Labeling of tOmpA with Alexa Fluor 488

Purified Cys-tOmpA was covalently labeled with Alexa Fluor 488 dye *via* maleimide chemistry. Alexa Fluor 488 C5 maleimide (Thermo Fisher Scientific, UK) dissolved in DMSO (10 mg/mL) was added to a sample containing 50 μM Cys-tOmpA, 6 M GuHCl, 0.5 mM TCEP, and 25 mM Tris–HCl (pH 7.2) to a final concentration of 0.5 mM. The total sample volume was 500 μL. The labeling reaction was left overnight at 4 °C and then loaded onto Superdex Peptide 10/300 column equilibrated with 6 M GuHCl and 25 mM Tris–HCl (pH 7.2) to remove the excess free dye. Samples were collected every 1 mL and peak protein fractions tested for dye labeling using a Nanodrop 2000 (Thermo Fisher Scientific, UK). The labeling efficiency was ~ 50%.

#### MST protocol

From a 200 μM SurA stock solution in 50 mM glycine–NaOH (pH 9.5), a series of two-fold serial dilutions were performed to obtain sixteen 15 μL samples. Labeled Cys-tOmpA was buffer exchanged into 8 M urea and 50 mM glycine–NaOH (pH 9.5) to a concentration of 1.6 μM. This stock was diluted 16-fold to a concentration of 100 nM with 50 mM glycine–NaOH (pH 9.5), then immediately added to the 16 SurA-containing samples in 15 μL aliquots (30-μL total sample volume). The final sample concentrations were 100 nM Cys-tOmpA, 100 μM–3 nM SurA, 0.25 M urea, and 50 mM glycine–NaOH (pH 9.5). Samples were rapidly added to capillaries by capillary action then read using a Monolith NT.115 MST machine (NanoTemper, München, Germany). To obtain the dissociation constant, *K*_d_, data were fitted to the Hill equation:

$$S_{\mathrm{obs}}=S_{U}+\left(S_{B}-S_{U}\right).\left(\frac{{\left[\text{SurA}\right]}^{n}}{K_{D}+{\left[\text{SurA}\right]}^{n}}\right)$$

where *S*_obs_ is the observed signal, *S*_U_ is the signal from unbound tOmpA, *S*_B_ is the signal from bound tOmpA, and *n* is the Hill coefficient. Data fitting was carried out using IgorPro 6.3.4.1 (Wavemetrics).

### Circular dichroism

Far-UV circular dichroism spectra of BamA folded in 100 nm LUVs composed of DLPC, DTPC, or DMPC were acquired on a Chirascan plus circular dichroism spectrometer (Applied PhotoPhysics) with a bandwidth of 2.5 nm, a scan speed of 0.5 nm s^− 1^, a step size of 1 nm, and a path length of 1.0 mm. The average of eight scans was taken to enhance signal to noise. Samples contained 1.5 μM BamA, 1.2 mM lipids (molar LPR 800:1), 0.24 M urea, and 50 mM glycine–NaOH (pH 9.5) at 30 °C and were pre-folded overnight at 30 °C. A molar LPR of 800:1 was used to reduce light scattering. Corresponding blank spectra, containing all reagents except BamA, were taken and subtracted for each sample. The mean residue ellipticity (MRE) at each wavelength was obtained by first calculating the mean residue weight (MRW):

$$\mathrm{MRW}=\frac{M}{N-1}$$

where *M* is the molecular mass of the protein in Daltons, and *N* is the number of amino acids it contains. The MRE is then given by:

$${\left[\theta \right]}_{\mathrm{MRE}}=\frac{\mathrm{MRW}\times \theta_{\lambda }\;}{10\times d\times c}$$

where [θ]_MRE_ is the MRE, θ_*λ*_ is the measured ellipticity at a particular wavelength, *d* is the path length in cm, and *c* is the concentration in g/mL.

### MD simulations

CG-MD simulations of the BamA barrel domain (tBamA) and the OmpA barrel domain (tOmpA) in membranes with different hydrophobic thicknesses were performed with GROMACS 5.0.2 [70] using the MARTINI22 force field [44], [71]. A CG-MD approach was selected to allow access to longer timescales than are typically available with all-atom simulations. A variety of studies have demonstrated that CG-MD simulations can replicate lipid behavior [44]. In the MARTINI force field, atoms are grouped into particles, which consist of ~ 4–5 atoms, enabling access to μs timescales [44], [72]. CG-MD simulations were performed in three different lipid types (*di*_C8:0–10:0_PC, *di*_C12:0–14:0_PC, and *di*_C16:0–18:0_PC), in which the acyl chains are represented by two, three, and four particles, respectively. An elastic network model was added with a cutoff distance of 0.7 nm to restrain the protein secondary and tertiary structures [73]. Note that while this prevents the possible observation of any barrel opening events, the cross-linking data in Fig. 4 demonstrate that the catalytic effect of BamA observed in thicker membranes is not dependent on lateral gating.

For the tBamA simulations, prior to the CG-MD simulations, a 50-ns atomistic simulation was performed using a full-length BamA model (from Ref. [74]) in a DMPC bilayer. The system was minimized (10,000 steps) followed by equilibration for 0.675 ns with gradual releasing of restraints prior to 50-ns unrestrained simulation. The system contained 539 DMPC lipids and was neutralized with 27 potassium ions. The pressure was maintained using a Nose–Hoover Langevin barostat [75], [76], and the temperature was maintained using a Langevin thermostat. The temperature of the system was 303.15 K and the timestep was 2 fs. The system was built using CHARMM-GUI [77]. The BamA barrel domain, residues 425–810, from the final frame of this simulation was used to generate the CG protein. The CG simulation systems for tBamA are shown in Fig. S8a–c. The tBamA simulations in *di*_C8:0–10:0_PC, *di*_C12:0–14:0_PC, and *di*_C16:0–18:0_PC bilayers contained 391, 369, and 375 lipids, respectively. For the tOmpA simulations, the structure was taken from PDB: 1QJP [78], with mutated residues in the structure replaced with wild-type residues and missing residues in the loops built in using MODELLER [79]. The tOmpA simulations in *di*_C8:0–10:0_PC, *di*_C12:0–14:0_PC, and *di*_C16:0–18:0_PC bilayers contained 393, 416, and 400 lipids, respectively.

Five CG-MD simulations of 3 μs were performed for each protein in each lipid type. NaCl ions at a concentration of 150 mM were used to neutralize the systems. All systems were minimized (5000 steps) followed by equilibration for 4.75 ns with gradual releasing of lipid restraints. Systems were initially built using CHARMM-GUI [77] prior to the addition of the elastic network. In all CG-MD simulations, the temperature of the systems was 323 K. The LINCS algorithm was used to constrain bond length to equilibrium lengths [80]. Lennard–Jones interactions were shifted to zero between 0.9 and 1.2 nm. Coulombic interactions were shifted to zero between 0 and 1.2 nm. A Berendsen barostat [81] with semi-isotropic conditions was used to control the pressure (to 1 bar) and a v-rescale thermostat was used to control the temperature. The timestep was 20 fs.

Scripts to analyze membrane thickness and order parameters made use of the MDAnalysis Python library [82]. To calculate < P_2_ > order parameters and membrane thickness for lipids in the vicinity of the tBamA β1–β16 seam or the opposite side of the barrel, for each frame, lipids were selected for analysis if they were within 12 Å of residues K808 or L613, respectively. For tOmpA, lipids were selected for analysis if they were within 12 Å of residues or L164 (β1–β18 seam) or L79 (opposite side of the barrel). For calculation of < P_2_ > order parameters and membrane thickness of bulk lipid, lipids were selected from each frame if the location of their phosphate beads was > 30 Å from the surface of tBamA or tOmpA. Order parameters for each acyl chain bond were calculated using:

$$S_{<P_{2}>}=0.5\times 〈\left(3\times \cos^{2}\theta \right)-1)〉$$

where *θ* is the angle between the bond and the bilayer normal [83], [84], [85].

## Appendix A Supplementary data

Supplementary methods, figures and tables. Image 2
