# Supplementary material for: Effects of Periplasmic Chaperones and Membrane Thickness on BamA-Catalyzed Outer-Membrane Protein Folding
Source: J Mol Biol. 2017 Nov 24;429(23):3776–92. doi: 10.1016/j.jmb.2017.09.008 (PMC5692476; doi:10.1016/j.jmb.2017.09.008)
Supplement: Supplementary file 1 — Supplementary methods, figures and tables. [file mmc1.docx]

**Supplementary Information**

# Effects of periplasmic chaperones and membrane thickness on BamA-catalysed outer membrane protein folding *in vitro*

**Bob Schiffrin^1^, Antonio N. Calabrese^1,3^, Anna J. Higgins^1,3^, Julia R. Humes^1^, Alison E. Ashcroft^1^, Antreas C. Kalli^2^, David J. Brockwell^1,*^, Sheena E. Radford^1,*^**

**^1^**Astbury Centre for Structural Molecular Biology, School of Molecular and Cellular Biology, Faculty of Biological Sciences, University of Leeds, LS2 9JT, UK.

**^2^**Astbury Centre for Structural Molecular Biology, Leeds Institute of Cancer & Pathology, University of Leeds, Wellcome Trust Brenner Building, St James’s University Hospital, LS9 7TF.

^3^These authors contributed equally to this work

*Corresponding authors. E-mail: d.j.brockwell@leeds.ac.uk or s.e.radford@leeds.ac.uk.

**Supplementary Methods**

**Plasmids**

The plasmids for tOmpA, OmpA and BamA which encode the mature OMP sequences in pET11a were kindly provided by Karen Fleming (John Hopkins University, USA) [1]. BamA^C690S/C700S^ (BamA^Cys-free^) and BamA^C690S/C700S/I430C/K808C^ (BamA^X-link^) were created from BamA-pET11a by site-directed mutagenesis using Q5 site-directed mutagenesis (NEB, UK). A tOmpA mutant containing an N-terminal cysteine residiue (Cys-tOmpA-pET11a) was made by Q5 site-directed mutagenesis (NEB, UK) of the tOmpA-pET11a plasmid. To create tBamA-pET11a, residues 425-810 of BamA were amplified by PCR, using plasmid BamAB-pETDUET-1 (kindly donated by Susan Buchanan (NIH, USA)) as the template, and the resultant product was then ligated into pET11a between NdeI and BamHI restriction sites. Skp-pET28b and SurA-pET28b plasmids were kindly provided by Sebastian Hiller (University of Basel, Switzerland) [2] and Daniel Kahne (Harvard University, USA), respectively. To create the expression plasmid for the BamA POTRA domains, a pMAL-c5X (NEB, UK) plasmid was modified by addition of an N-terminal 6x His-tag (HT) and replacement of the thrombin cleavage site with a TEV cleavage site. This vector was used to make a construct that contained maltose-binding protein (MBP) fused to BamA POTRA domains 1-5 of mature BamA (residues 21-425) separated by a TEV cleavage site (HT-MBP-TEV-POTRAs-pMAL).

**Expression and purification of tOmpA, Cys-tOmpA, tBamA, BamA, BamA^Cys‑free^, BamA^X-link^ and OmpA**

The relevant plasmid was transformed into BL21[DE3] cells (Stratagene, UK) and grown in 500 mL LB medium containing 100 µg/mL carbenicillin at 37 °C with shaking (200 rpm). Expression was induced with 1 mM IPTG when the culture reached an OD_600_ of 0.5-0.6, and then harvested after 4h by centrifugation (5000 *g*, 15 min, 4 °C). Cells were resuspended in 20 mL 50 mM Tris-HCl, pH 8.0, 5 mM EDTA, 1 mM phenylmethylsulfonyl fluoride (PMSF) , 2 mM benzamidine, and lysed by sonication (6 x 1 min bursts with 1 min cooling on ice between each sonication). The insoluble fraction was collected by centrifugation (25000 *g*, 30 min, 4 °C), resuspended in 20 mL 50 mM Tris-HCl, pH 8.0, 2 % (*v/v)* Triton-X-100 and incubated for 1 h at room temperature, with gentle agitation. The insoluble fraction was again pelleted (25000 *g*, 30 min, 4 °C) and the inclusion bodies were washed twice by resuspending in 50 mM Tris-HCl, pH 8.0, incubating for 1 h at room temperature with gentle agitation, followed by centrifugation (25000 *g*, 30 min, 4 °C). The inclusion bodies were solubilised in 25 mM Tris-HCl, 6 M Gdn-HCl, pH 8.0 and centrifuged (20,000 *g*, 20 min, 4 ºC)**.** The supernatant was filtered (0.2 μM syringe filter, Sartorius, UK) and purified further by gel filtration in 25 mM Tris-HCl, 6 M Gdn-HCl, pH 8.0. Peak fractions were concentrated to ~500 μM using Vivaspin 20 (5 kDa MWCO) concentrators (Sartorius, UK), snap-frozen in liquid nitrogen, and stored at -80 °C. Protein concentrations were determined spectrophotometrically using calculated molar extinction coefficients at 280 nm of 46,870, 101,315, 140,165, 140,040, 140,165 and 52,955 and M^-1^ cm^-1^ for tOmpA, tBamA, BamA, BamA^Cys‑free^, BamA^X-link^ and OmpA, respectively [3].

## Cloning, expression and purification of BamA POTRA domains

The HT-MBP-TEV-POTRAs-pMAL vector (see Plasmids above) was transformed into BL21[DE3] cells, and a single colony used to inoculate 10 mL LB starter cultures containing 100 μg/ml carbenicillin which were grown overnight (37 ºC, 200 rpm). The starter cultures were used to inoculate 2x 1 L of LB, supplemented with 100 μg/ml carbenicillin. Cells were grown at 37 °C with shaking (200 rpm) until the culture reached an OD_600_ of ~0.6, at which point protein expression was induced with 1 mM IPTG. After 4 h expression cells were harvested by centrifugation (5000 *g*, 15 min, 4 °C). Cells were resuspended in 20 mM Tris-HCl, pH 8.0, 300 mM NaCl, 20 mM imidazole, EDTA-free protease inhibitor tablets (Pierce Biotechnology, USA), and lysed using a cell disrupter (Constant Cell Disruption Systems, UK). Following centrifugation to remove cell debris (20 min, 4 °C, 39000 *g*), the lysate was applied to 4x 5 ml HisTrap columns and washed with 20 mM Tris-HCl, pH 8.0, 300 mM NaCl, 20 mM imidazole. HT-MBP-TEV-POTRAs was eluted with a 0-500 mM imidazole gradient with 20 mM Tris-HCl, pH 8.0, 300 mM NaCl. Fractions containing HT-MBP-TEV-POTRAs were pooled and dialysed into 20 mM Tris-HCl, pH 8.0, 300 mM NaCl, and diluted to ~100 ml with 20 mM Tris-HCl, pH 8.0, 300 mM NaCl to prevent aggregation during TEV cleavage. Following addition of 14.4 mM β-mercaptoethanol (BME), His-tagged TEV protease (see below) was added at a 1:10 TEV: HT-MBP-TEV-POTRAs molar ratio and incubated overnight at 4 ºC. The cleavage reaction was passed over 4x 5 ml HisTrap columns and the flow through, containing cleaved BamA POTRA domains was collected. BamA POTRA domains were concentrated to ~100 μM using Vivaspin 20 (5 kDa MWCO) concentrators (Sartorius, UK), aliquoted, snap-frozen in liquid nitrogen and stored at -80 ^°^C. Protein concentration was determined spectrophotometrically using a calculated molar extinction coefficient at 280 nm of 38,850 M^-1^ cm^-1^ [3].

**Expression and purification of His-tagged Skp**

His-tagged Skp was expressed and purified using a protocol adapted from Burmann *et al.* [2].The pET28b plasmid, containing the Skp gene with an N-terminal 6xHis-tag and thrombin cleavage site, was transformed into BL21[DE3]pLysS cells (Stratagene, UK). Cells were grown in LB medium containing 30 µg/mL kanamycin at 37 °C with shaking (200 rpm) until the culture reached an OD_600_ of ~0.6. The temperature was then lowered to 20 °C and protein expression induced with 0.4 mM IPTG. Following overnight expression (~18 h) cells were harvested by centrifugation, resuspended in 25 mM Tris-HCl, pH 7.2, 150 mM NaCl, 20 mM imidazole, containing a cocktail of EDTA-free protease inhibitors (Roche), and lysed using a cell disrupter (Constant Cell Disruption Systems, UK). Following centrifugation to remove cell debris (20 mins, 4 °C, 39000 *g*), the lysate was applied to 4x 5 mL HisTrap columns (GE Healthcare) and washed with 25 mM Tris-HCl, pH 7.2, 150 mM NaCl, 20 mM imidazole. His-tagged Skp was denatured on-column with 25 mM Tris-HCl, 6 M Gdn-HCl, pH 7.2, and eluted with a 0-500 mM imidazole gradient over 50 mL in 25 mM Tris-HCl, 6 M Gdn-HCl, pH 7.2. Fractions containing Skp were pooled and the protein refolded by dialysis against 25 mM Tris-HCl, pH 7.2, 150 mM NaCl. Refolded His-tagged Skp was concentrated to ~50 µM (trimer) using Vivaspin 20 (5 kDa MWCO) concentrators (Sartorius, UK), aliquoted, snap-frozen in liquid nitrogen and stored at -80 ^°^C. Protein concentrations were determined using a bicinchoninic acid (BCA) assay (Thermo Fisher Scientific, UK), according to the manufacturer’s instructions.

**Expression and purification of His-tagged SurA**

His-tagged SurA was expressed from a pET28b plasmid containing the SurA gene with an N-terminal 6xHis-tag and thrombin cleavage site. His-tagged SurA was expressed and purified using the same protocol as for His-tagged Skp (above) and stored at a concentration of ~200 µM. Protein concentration was determined spectrophotometrically using a calculated molar extinction coefficient at 280 nm of 29,450 M^-1^ cm^-1^ [3].

## Expression and purification of His-tagged TEV protease

Vector pMHTDelta238 containing His-tagged TEV fused with MBP which is removed *in vivo* by autocleavage [4], was obtained from DNASU (Clone TvCD00084286). This vector was transformed into BL21-CodonPlus[DE3]-RIPL cells (Stratagene, UK). Cells were grown in LB medium containing 50 µg/mL kanamycin at 37 °C with shaking (200 rpm) until the culture reached an OD_600_ of ~0.6. The temperature was then lowered to 30 °C and expression induced with 0.5 mM IPTG. Following expression (~4 h) cells were harvested by centrifugation, resuspended in 25 mM sodium phosphate buffer, pH 8.0, 200 mM NaCl, 10% (*v/v*) glycerol, 25 mM imidazole, 1 mM PMSF, 2 mM benzamidine, ~0.02 mg/ml DNase (Sigma, UK), and lysed by sonication (6 x 30 s bursts with 1 min cooling on ice between each sonication). Following centrifugation to remove cell debris (20 mins, 4 °C, 39000 *g*), the lysate was applied to Ni^2+^ Sepharose beads (GE Healthcare) and washed twice with 25 mM sodium phosphate buffer, pH 8.0, 200 mM NaCl, 10% (*v/v*) glycerol, 25 mM imidazole. His-tagged TEV was eluted with 25 mM sodium phosphate buffer, pH 8.0, 200 mM NaCl, 10% (*v/v*) glycerol, 500 mM imidazole. The eluate was filtered (0.2 μM syringe filter, Sartorius, UK) and gel filtered on a HiLoad Superdex 75 26/60 column (GE Heallthcare) equilibrated with 25 mM sodium phosphate buffer, pH 8.0, 200 mM NaCl, 25 mM imidazole, 10% (*v/v*) glycerol, 5 mM β-mercaptoethanol. Peak fractions were concentrated to ~1 mg/mL using Vivaspin 20 (5 kDa MWCO) concentrators (Sartorius, UK), aliquoted, snap-frozen in liquid nitrogen and stored at ‑80 ^°^C.


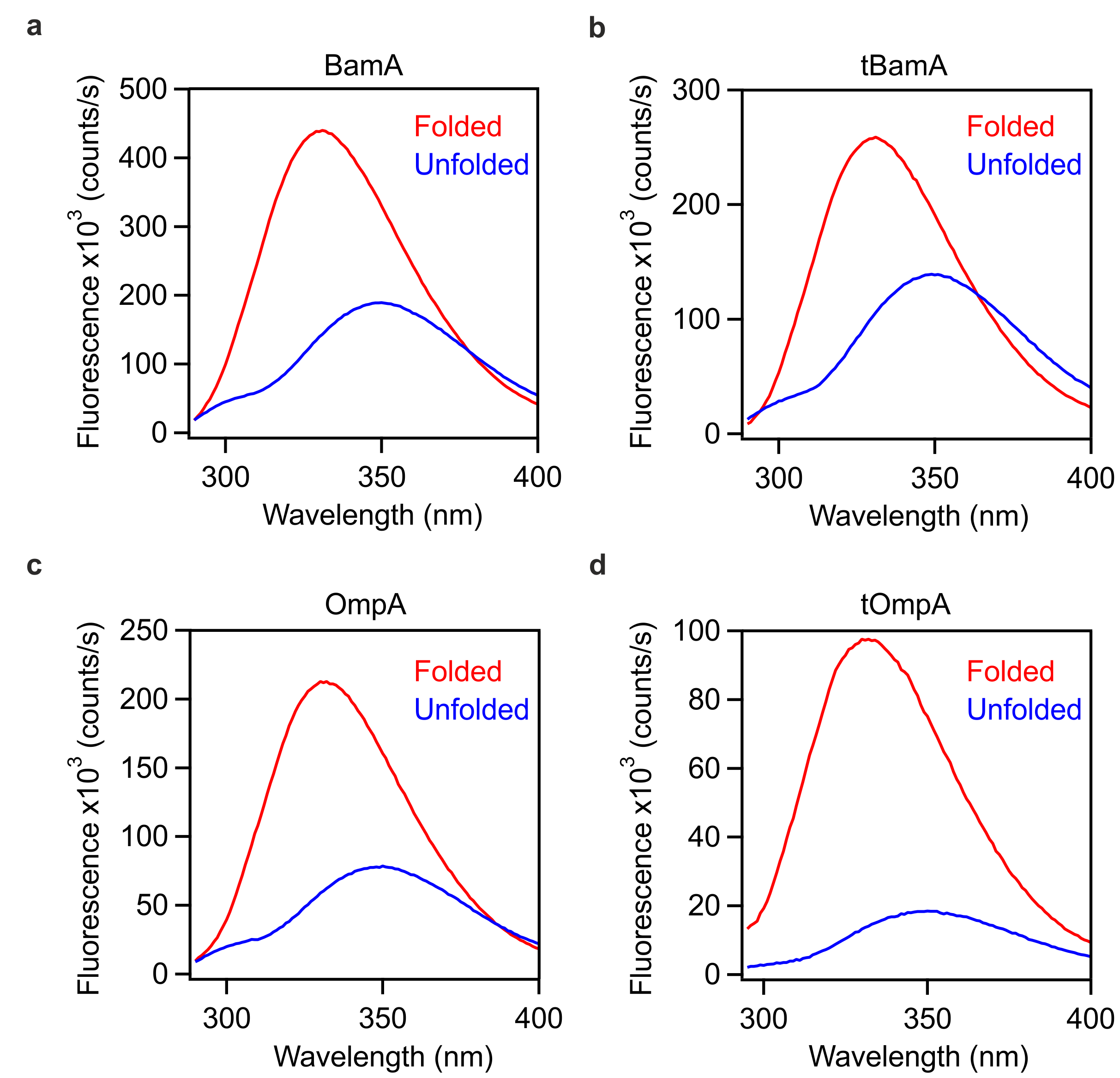


**Fig. S1.** Fluorescence emission spectra of folded and unfolded OMPs. (a) BamA, (b) tBamA, (c) OmpA, and (d) tOmpA. Folded samples (red) contained 0.24 M urea, 1.28 mM DUPC LUVs, 50 mM glycine-NaOH, pH 9.5, and were incubated for >1.5 h following initiation of the folding reaction. Unfolded samples (blue) contained 8 M urea, 50 mM glycine-NaOH, pH 9.5. Fluorescence was excited at 280 nm and emission measured between 290-400 nm. The OMP concentrations were 0.8 μM in (a*-*c) and 0.4 μM in (d). All spectra were acquired at 25 ºC.


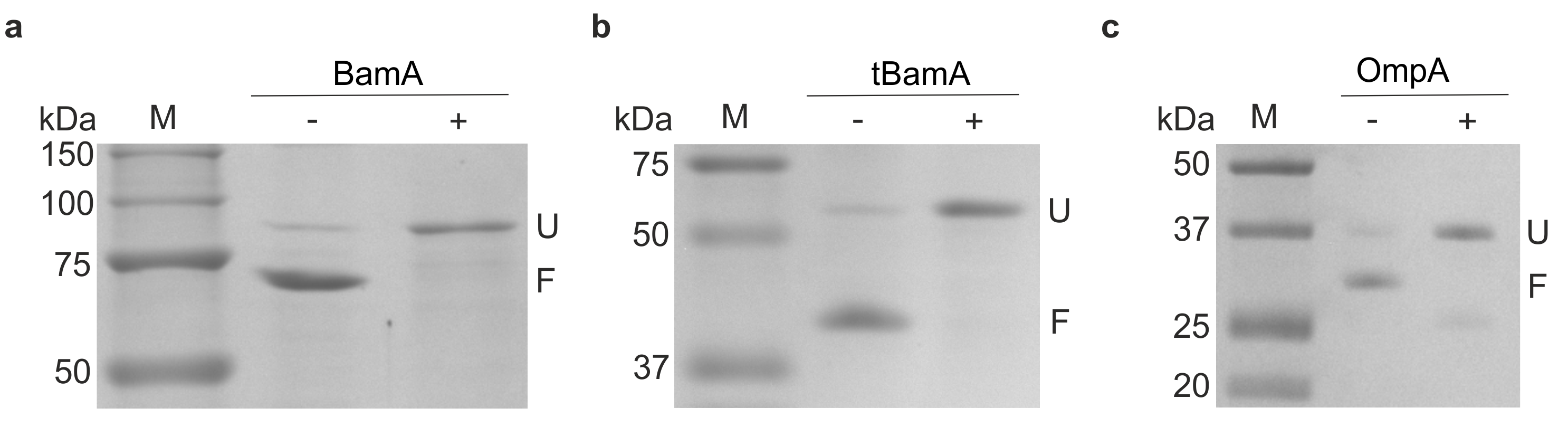


**Fig. S2.** BamA, tBamA and OmpA fold with similar yields into DUPC liposomes. Example SDS-PAGE band shift assays for (a) BamA, (b) tBamA, and (c) OmpA. For BamA and tBamA, the samples were run under semi-native conditions (see Methods). Samples contained 0.8 μM OMP, 0.24 M urea, 1.28 mM DUPC liposomes, in 50 mM glycine-NaOH, pH 9.5. The OMP concentrations and experimental conditions are the same as used in the kinetic experiments. The extent of OMP folding, quantified by densitometry, was 89.0 ± 2.0, 88.2 ± 1.2, and 85.4 ± 1.2 % for BamA, tBamA, and OmpA, respectively. These data are the mean ± the standard deviation of the folding yields obtained from three separate experiments, each using an independently prepared liposome batch. M: Marker lane. +/-: with or without boiling prior to gel loading. U: Unfolded. F: Folded.


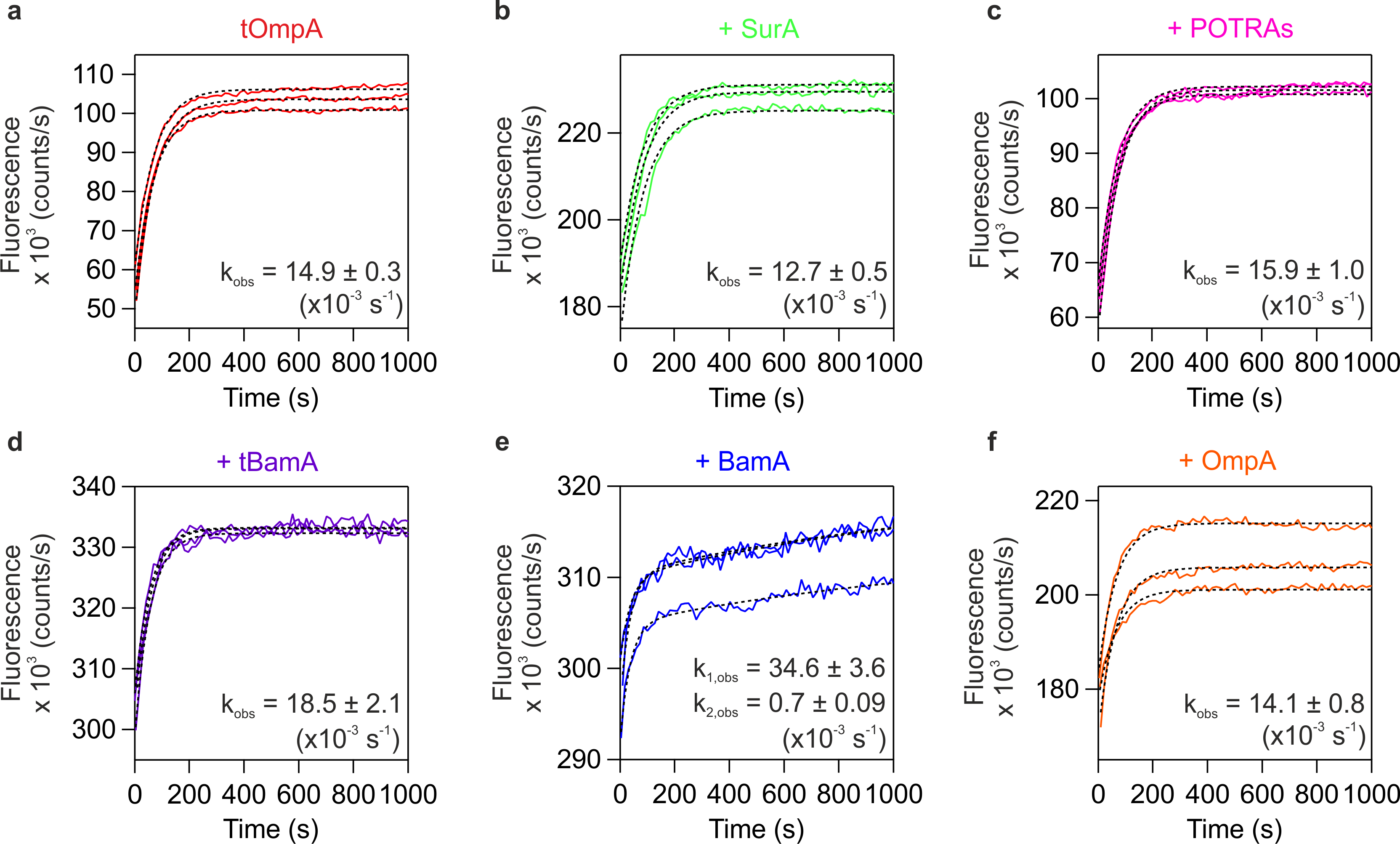


**Fig. S3.** Effects of BamA and its constituent domains on the folding kinetics of tOmpA in DUPC liposomes. Kinetic folding traces for (a) tOmpA alone, and in the presence of (b) SurA, (c) BamA POTRA domains, (d) prefolded tBamA, (e) prefolded BamA (full-length), and (f) prefolded OmpA. Samples contained 0.4 μM tOmpA, 1.28 mM DUPC, 0.24 M urea, 50 mM glycine-NaOH, pH 9.5, at 25 ºC. A two-fold molar excess (0.8 μM) of SurA, BamA POTRA domains, tBamA, BamA or OmpA was used. Three transients are shown in each panel. Fits to a single exponential, (a-d and f), and double exponential function, (e), are indicated by dashed black lines.


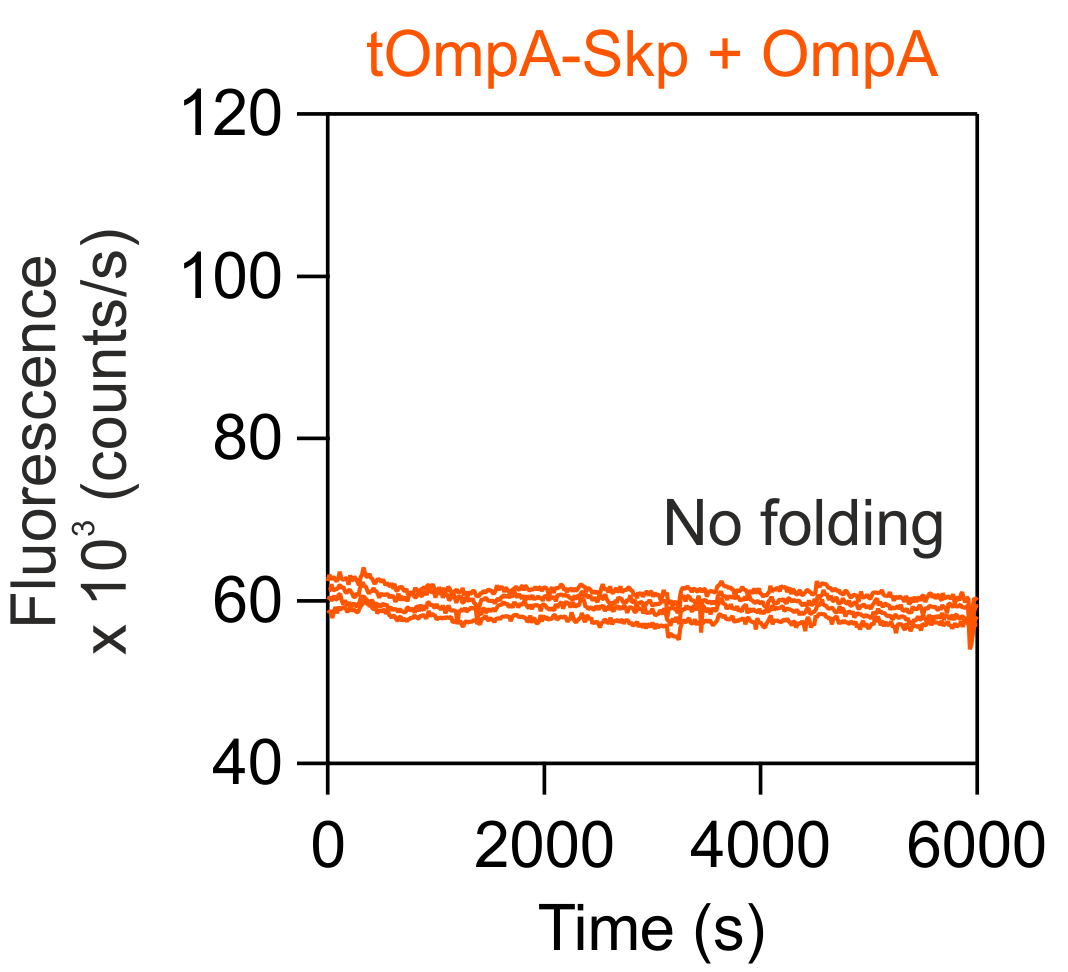


**Fig. S4.** No folding is observed on addition of Skp-tOmpA to DUPC liposomes containing prefolded OmpA. Samples contained 0.4 μM tOmpA, 0.8 μM Skp, 0.8 μM OmpA (prefolded), 1.28 mM DUPC, 0.24 M urea, 50 mM glycine-NaOH, pH 9.5, at 25 ºC. tOmpA was pre-incubated with Skp, then added to OmpA prefolded into DUPC LUVs (see Methods).


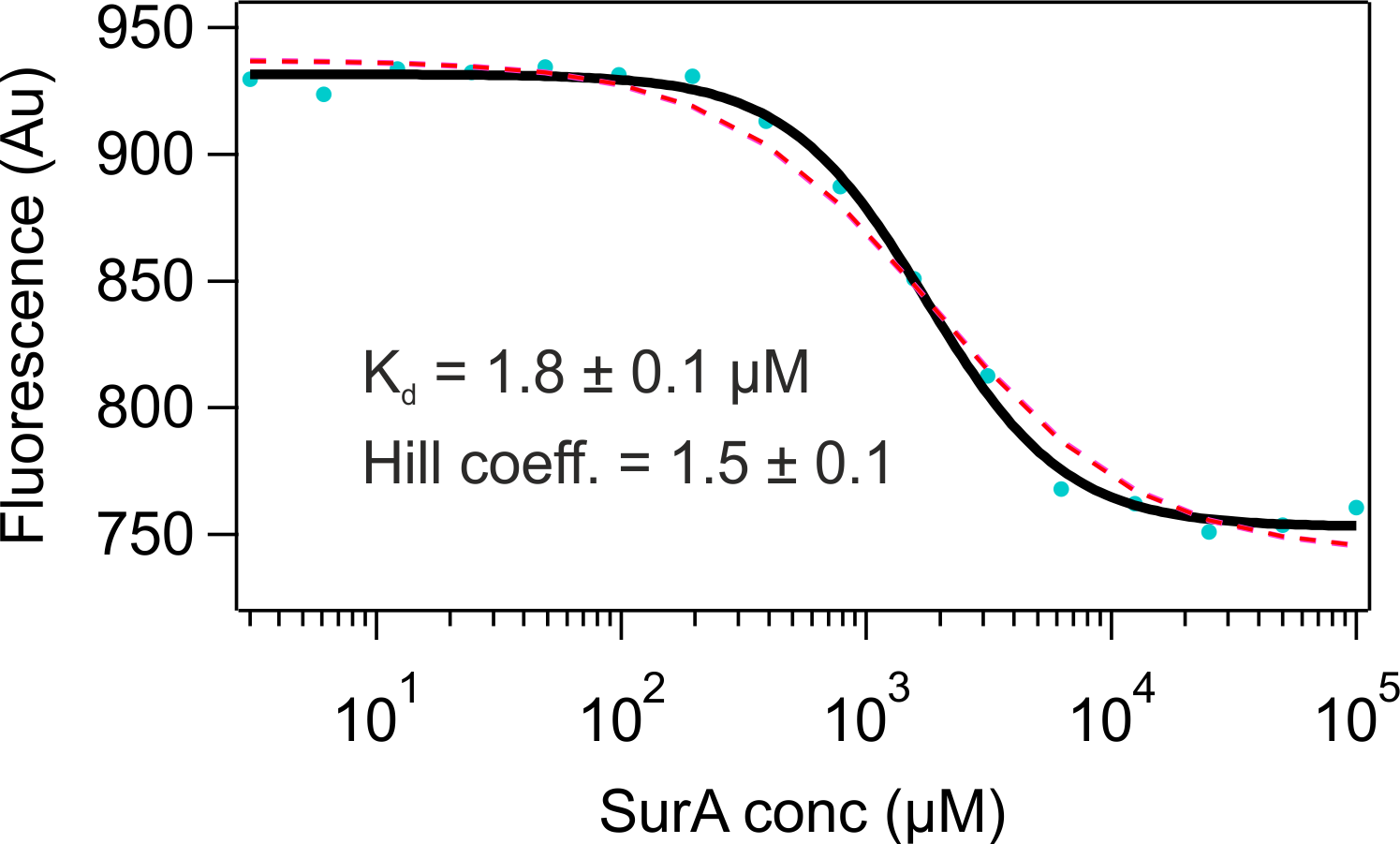


**Fig. S5.** Example microscale thermophoresis (MST) data for binding of SurA to tOmpA. Samples contained 100 nM Alexa Fluor 488-labelled tOmpA (see Methods), SurA (0.3 nM-100 μM), 0.24 M urea, 50 mM glycine-NaOH, pH 9.5. A fit to the Hill equation is indicated by a black solid line. The K_d_ value (1.8 ± 0.1 μM) and Hill coefficient values (1.5 ± 0.1) are the mean ± standard deviation of three independent experiments. The data are poorly fit to a Hill equation in which the Hill coefficient is forced to take a value of 1 (dashed pink line), suggesting a greater than 1:1 SurA:tOmpA stoichiometry.


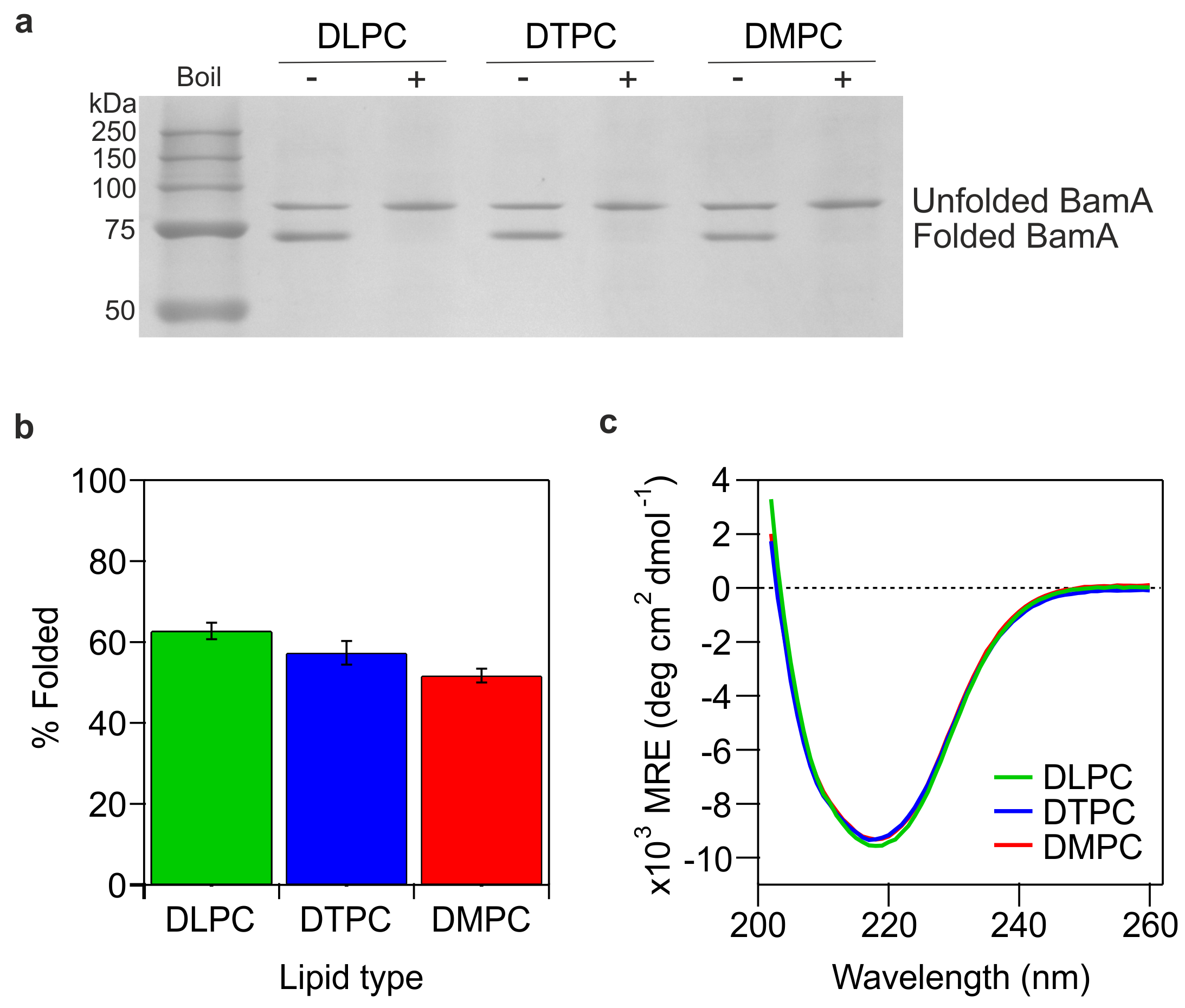


**Fig. S6.** BamA folds with similar yields into DLPC, DTPC and DMPC liposomes. (a) Semi-native SDS-PAGE band shift assay of BamA folding in DLPC (C12), DTPC (C13) and DMPC (C14) liposomes. M: Marker lane. +/-: with or without boiling prior to gel loading. U: Unfolded. F: Folded. (b) Quantification of data in (a) by densitometry. Error bars represent the standard deviation from three independent experiments. (c) CD spectra of BamA in DLPC, DTPC and DMPC LUVs. Samples for semi-native SDS-PAGE contained 0.8 μM BamA (the same concentration as used in kinetic assays), 1.28 mM lipids, 0.24 M urea, 50 mM glycine-NaOH, pH 9.5. Samples for CD contained 1.5 μM BamA, 1.2 mM lipids, 0.24 M urea, 50 mM glycine-NaOH, pH 9.5. A lower molar LPR was used in CD experiments to reduce the scattering effects from liposomes. All samples were folded overnight at 30 ºC.


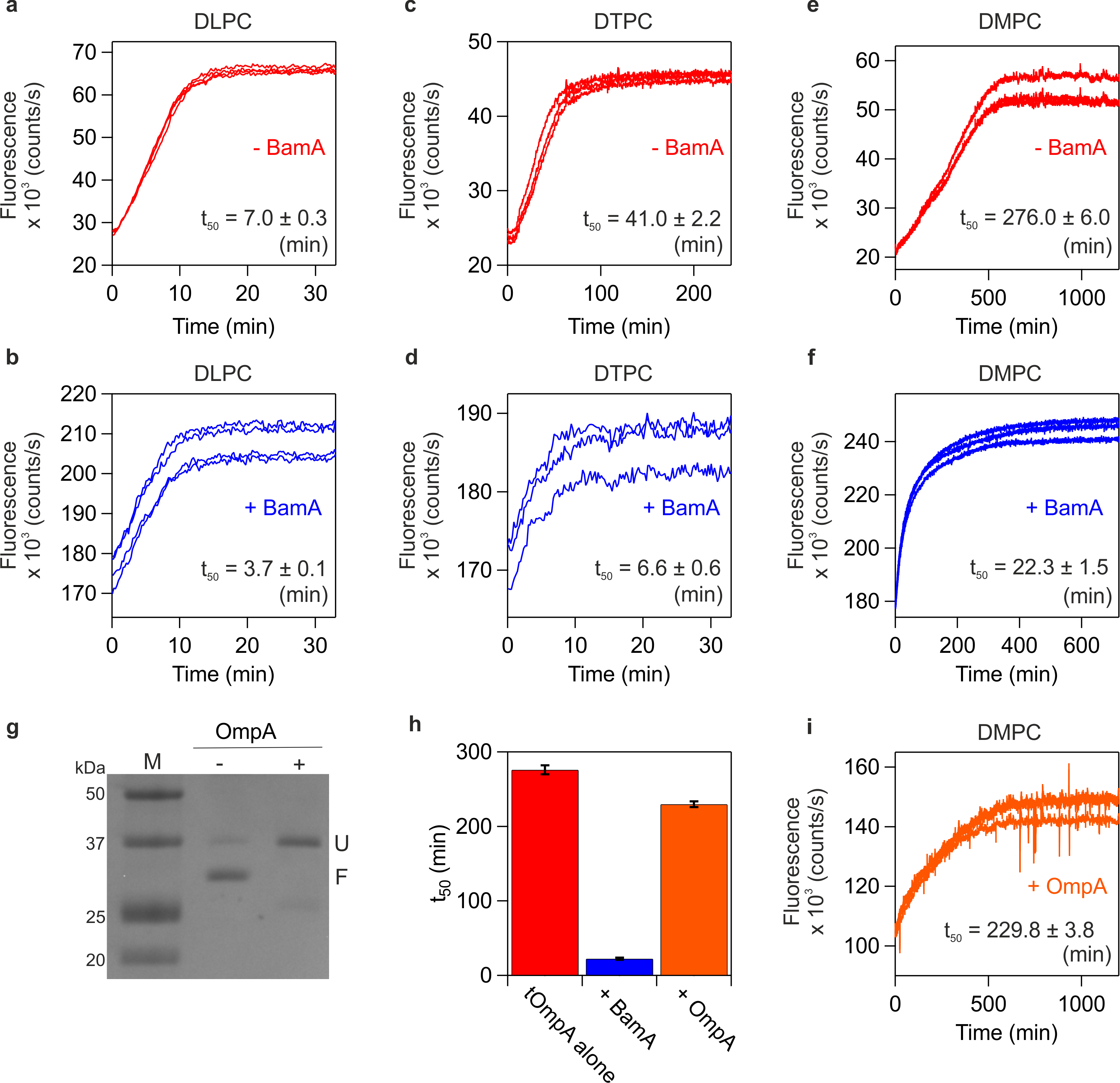


**Fig. S7.** Example raw kinetic traces of tOmpA folding in the presence or absence of BamA or OmpA, in DLPC, DTPC or DMPC liposomes. (a-f) Folding kinetics of tOmpA alone (upper) or in the presence of prefolded BamA (middle) into liposomes composed of (a,b) DLPC, (c,d) DTPC, or (e,f) DMPC. (g) SDS-PAGE band shift assay of OmpA folding in DMPC liposomes. The folding yield measured by densitometry was 88.1 ± 7.0 %. M: Marker lane. +/-: with or without boiling prior to gel loading. U: Unfolded. F: Folded. Samples contained 0.8 μM OmpA (the same concentration as used in kinetic assays), 1.28 mM DMPC, 0.24 M urea, 50 mM glycine-NaOH, pH 9.5, and were folded overnight at 30 ºC. (h) Comparison of t_50_ values for tOmpA folding into DMPC liposomes alone (red), and in the presence of prefolded BamA (blue) or OmpA (orange). (i) Folding kinetics of tOmpA in the presence of prefolded OmpA into liposomes composed of DMPC. Samples in (a-g,i) contained 0.4 μM tOmpA, 1.28 mM lipid, 0.24 M urea, 50 mM glycine-NaOH, pH 9.5. A two-fold molar excess (0.8 μM) of BamA or OmpA was folded overnight into liposomes overnight prior to addition of tOmpA. These experiments were performed at 30 ºC to ensure the temperature used was above the *T_m_* for DMPC (24 ºC) [5].


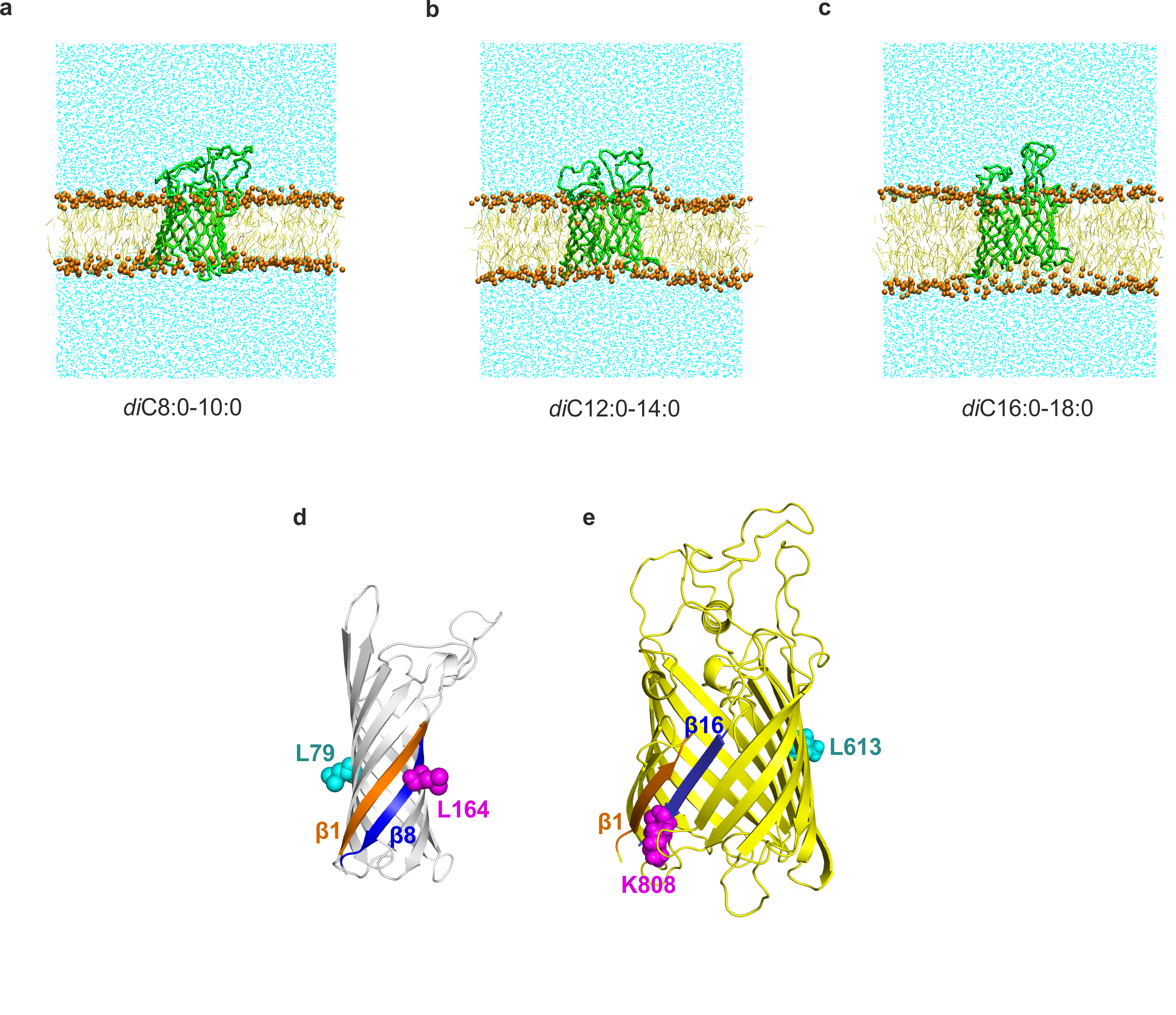


**Fig. S8.** Coarse-grained simulation systems of the BamA barrel in bilayers of different hydrophobic thicknesses. (a-c) Snapshot of tBamA CG-MD simulation systems at the beginning of the simulation. Acyl chains in the simulations are represented by (a) 2 particles (*di*_C8:0-10:0_PC), (b) 3 particles (*di*_C12:0‑14:0_PC), and (c) 4 particles (*di*_C16:0‑18:0_PC). Particles representing phosphate lipid head groups and lipid acyl chains are shown in orange and yellow, respectively. tBamA and water particles are shown in green and cyan, respectively. (d) Starting tOmpA model for the CG simulations. Structure was taken from PDB: 1QJP [6], with mutated residues in the structure replaced with wild-type residues and missing residues in the loops built in using MODELLER [7]. Strands β1 and β8 are coloured orange and blue, respectively. Residues L79 (β4) and L164 (β8) are highlighted as cyan and magenta spheres, respectively (e) Starting tBamA model for the CG simulations, following 50 ns of atomistic simulation in a DMPC bilayer (see Methods). Strands β1 and β16 are coloured orange and blue, respectively Residues L613 (β10) and K808 (β16) are highlighted as cyan and magenta spheres, respectively.

**
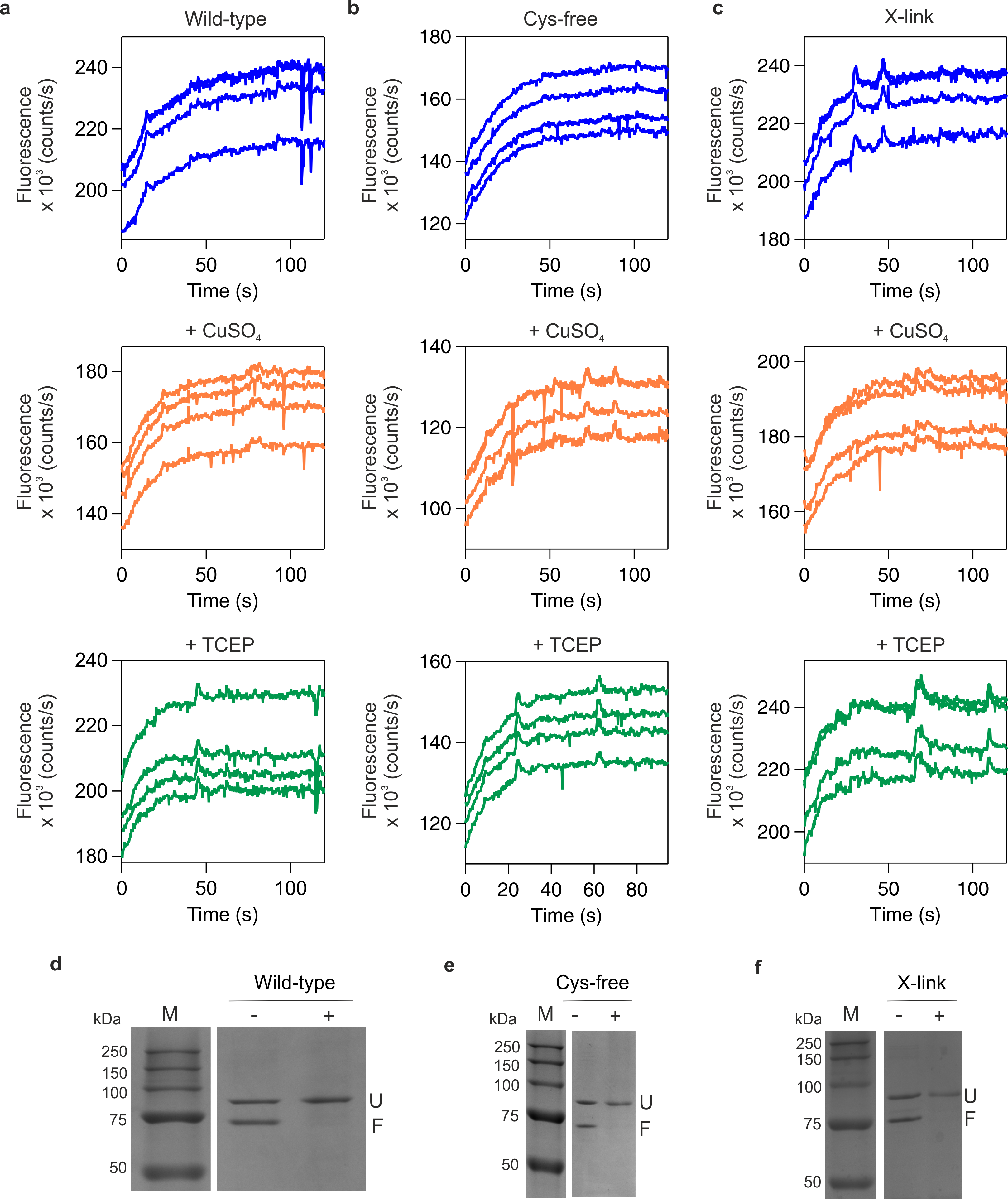
**

**Fig. S9**. Example raw kinetic traces of tOmpA folding into DMPC LUVs in the presence of BamA or BamA mutants, in the presence or absence of oxidising or reducing agents. Folding kinetics of tOmpA in the presence of (a) BamA (Wild-type), (b) BamA^Cys-free^, or (c) BamA^X-link^. Experiments were performed with no additions (upper, blue), or with addition of 1 mM CuSO_4_ (middle, orange) or 25 mM TCEP (lower, green). Samples contained 0.4 μM tOmpA, 1.28 mM DMPC, 0.24 M urea, 50 mM glycine-NaOH, pH 9.5. A two-fold molar excess (0.8 μM) of wild-type BamA, BamA^Cys‑free^ or BamA^X-link^ was used. Four transients for each of three liposome batches (12 in total) were used for calculation of the average t_50_ value for each condition (**Table S10**). These experiments were performed at 30 ºC to be above the *T_m_* for DMPC (24 ºC) [5]. (d-f) Example semi-native SDS-PAGE band shift assays for (d) wild-type BamA, (e) BamA^Cys-free^, and (f) BamA^X-link^. Samples contained 0.8 μM OMP, 0.24 M urea, 1.28 mM DMPC liposomes, in 50 mM glycine-NaOH, pH 9.5. The OMP concentrations and experimental conditions are the same as used in kinetic experiments. The fraction of OMP folded measured by densitometry for wild-type BamA, BamA^Cys-free^ or BamA^X-link^ were 51.7 ± 1.7, 55.9 ± 17.4, and 51.5 ± 11.9 %, respectively. These data are the mean ± the standard deviation of the folding yields obtained from three separate folding experiments, each using an independently prepared liposome batch. M: Marker lane. +/-: with or without boiling prior to gel loading. U: Unfolded. F: Folded.

| Folding reaction | k_1,obs_ (x10^-3^ s^-1^) | k_2,obs_ (x10^-3^ s^-1^) |
| --- | --- | --- |
| tOmpA alone | 14.9 ± 0.3 | N/A |
| tOmpA + SurA | 12.7 ± 0.5 | N/A |
| tOmpA + POTRAs | 15.9 ± 1.0 | N/A |
| tOmpA + tBamA | 18.5 ± 2.1 | N/A |
| tOmpA + BamA | 34.6 ± 3.6 | 0.7 ± 0.09 |
| tOmpA + OmpA | 14.1 ± 0.8 | N/A |

**Table S1.** Observed rate constants for tOmpA folding into DUPC liposomes in the presence of SurA, BamA constructs or prefolded OmpA. Samples contained 0.4 μM tOmpA, 1.28 mM DUPC, 0.24 M urea, 50 mM glycine-NaOH, pH 9.5, at 25 ºC. A two-fold molar excess (0.8 μM) of SurA, BamA POTRA domains, tBamA, BamA and OmpA was used. Data are shown as the mean ± the standard error of the mean (s.e.m.) of the rate constants obtained from three separate folding experiments, each using independently prepared batches of liposomes. For each batch of liposomes at least three folding transients were fitted globally to obtain the rate constants shown. N/A: The kinetic traces for the condition were adequately described by a single exponential.

| Folding reaction | k_obs_ (x10^-3^ s^-1^) |
| --- | --- |
| tOmpA-Skp | No folding |
| tOmpA-Skp + SurA | No folding |
| tOmpA-Skp + BamA | 1.2 ± 0.1 |
| tOmpA-Skp + tBamA | 0.39 ± 0.03 |
| tOmpA-Skp + POTRAs | No folding |
| tOmpA-Skp + OmpA | No folding |

**Table S2.** Observed rate constants for tOmpA folding into DUPC liposomes from its complex with Skp in the presence of SurA, BamA constructs, or prefolded OmpA. Samples contained 0.4 μM tOmpA, 1.28 mM DUPC, 0.24 M urea, 50 mM glycine-NaOH, pH 9.5, at 25 ºC. A two-fold molar excess (0.8 μM) of Skp, SurA, BamA POTRA domains, tBamA, BamA and OmpA was used. Data are shown as the mean ± the standard error of the mean (s.e.m.) of the rate constants obtained from three separate folding experiments, each using independently prepared batches of liposomes. For each batch of liposomes at least three folding transients were fitted globally to obtain the rate constants shown.

| **Protein or complex** | **Expected mass (Da)** | **Observed mass (Da)** |
| --- | --- | --- |
| SurA | 47241.3 | 47238.0 ± 5.5 |
| Skp | 53958.9 | 53961.3 ± 6.2 |
| Skp-tOmpA | 72833.8 | 72827.9 ± 7.4 |
| 1:1 SurA-tOmpA | 66116.2 | 66118.9 ± 5.1 |
| 2:1 SurA-tOmpA | 113357.5 | 113392.7 ± 3.2 |

Table S3: Observed and expected masses for native-MS data for interactions between Skp, SurA, and tOmpA. Raw data are shown in Fig. 2. The errors shown are the standard deviations of the mass measurements.

| **Lipid type** | **Acyl chain length** | **BamA folding yield (%)** |
| --- | --- | --- |
| DLPC | C12 | 62.7 ± 2.0 |
| DTPC | C13 | 57.3 ± 2.9 |
| DMPC | C14 | 51.7 ± 1.7 |

**Table S4.** BamA folding yields in DLPC, DTPC and DMPC liposomes. Folding yields were determined by densitometry following semi-native SDS-PAGE analysis (see Methods). Samples contained 0.8 μM BamA, 1.28 mM lipids, 0.24 M urea, 50 mM glycine-NaOH, pH 9.5, and were folded overnight at 30 ºC. Data shown are the mean ± the standard deviation of the folding yields obtained from three separate folding experiments, each using an independently prepared liposome batch.

| Lipid type | Folding reaction | t_50_ value (min) | Fold change  (+/- BamA/OmpA) |
| --- | --- | --- | --- |
| DLPC | tOmpA alone | 7.0 ± 0.3 | - |
|  | tOmpA + BamA | 3.7 ± 0.1 | 1.9 ± 0.1 |
| DTPC | tOmpA alone | 41.0 ± 2.2 | - |
|  | tOmpA + BamA | 6.6 ± 0.6 | 6.2 ± 0.6 |
| DMPC | tOmpA alone | 276.0 ± 6.0 | - |
|  | tOmpA + BamA | 22.3 ± 1.5 | 12.4 ± 0.9 |
|  | tOmpA + OmpA | 229.8 ± 3.8 | 1.2 ± 0.4 |

**Table S5.** Measured t_50_ values for tOmpA folding into DLPC, DTPC or DMPC liposomes in the presence or absence of BamA or OmpA. Samples contained 0.4 μM tOmpA, 1.28 mM lipid, 0.24 M urea, 50 mM glycine-NaOH, pH 9.5, at 30 ºC. In BamA- or OmpA-containing samples, a two-fold molar excess (0.8 μM) of BamA or OmpA was used. Data are shown as the mean ± the standard error of the mean (s.e.m.) of the t_50_ values obtained from three separate folding experiments, each using independently prepared batches of liposomes. Fold change errors are the propagated s.e.m. from folding reactions in the presence or absence of BamA.

| **Lipid type** | **Bond** | **Lipid region** | **<P_2_> order parameter** |
| --- | --- | --- | --- |
| *di*C8:0-10:0 | G-1 | Bulk | 0.48 ± 0.0003 |
|  | G-1 | L613 | 0.36 ± 0.08 |
|  | G-1 | K808 | 0.33 ± 0.09 |
|  | 1-2 | Bulk | 0.35 ± 0.0003 |
|  | 1-2 | L613 | 0.24 ± 0.006 |
|  | 1-2 | K808 | 0.23 ± 0.005 |
| *di*C12:0-14:0 | G-1 | Bulk | 0.51 ± 0.0003 |
|  | G-1 | L613 | 0.38 ± 0.01 |
|  | G-1 | K808 | 0.28 ± 0.02 |
|  | 1-2 | Bulk | 0.40 ± 0.0006 |
|  | 1-2 | L613 | 0.27 ± 0.009 |
|  | 1-2 | K808 | 0.22 ± 0.01 |
|  | 2-3 | Bulk | 0.37 ± 0.0006 |
|  | 2-3 | L613 | 0.24 ± 0.009 |
|  | 2-3 | K808 | 0.19 ± 0.01 |
| *di*C16:0-18:0 | G-1 | Bulk | 0.52 ± 0.0001 |
|  | G-1 | L613 | 0.39 ± 0.01 |
|  | G-1 | K808 | 0.22 ± 0.01 |
|  | 1-2 | Bulk | 0.48 ± 0.0002 |
|  | 1-2 | L613 | 0.31 ± 0.01 |
|  | 1-2 | K808 | 0.21 ± 0.01 |
|  | 2-3 | Bulk | 0.39 ± 0.0003 |
|  | 2-3 | L613 | 0.25 ± 0.01 |
|  | 2-3 | K808 | 0.16 ± 0.008 |
|  | 3-4 | Bulk | 0.34 ± 0.0005 |
|  | 3-4 | L613 | 0.20 ± 0.01 |
|  | 3-4 | K808 | 0.12 ± 0.008 |

**Table S6.** <P_2_> bond order parameters of lipids from coarse-grained molecular dynamics simulations of tBamA in bilayers of different thicknesses. Data are shown for bulk lipids (those greater than 30 Å from tBamA), and those within 12 Å of K808, at the β1-β16 seam of tBamA, and L613 on the opposite side of the barrel (**Fig. S8e**). Order parameters values are shown for the bonds between glycerol particles and first lipid acyl chain particles (G-1), and between consecutive lipid acyl chain particles (1-2, 2-3, and 3-4). Data shown are the mean ± standard deviation from 5 independent simulations. The duration of each simulation was 3 μs and data analysis was performed on the final 2.5 μs.

| **Lipid type** | **Lipid region** | **Membrane thickness (Å)** |
| --- | --- | --- |
| *di*C8:0-10:0 | Bulk | 25.8 ± 0.5 |
|  | L613 | 21.9 ± 0.8 |
|  | K808 | 22.2 ± 0.4 |
| *di*C12:0-14:0 | Bulk | 34.0 ± 0.2 |
|  | L613 | 27.6 ± 0.3 |
|  | K808 | 25.6 ± 0.2 |
| *di*C16:0-18:0 | Bulk | 40.9 ± 0.1 |
|  | L613 | 32.2 ± 0.5 |
|  | K808 | 28.0 ± 0.3 |

**Table S7.** Membrane thickness of lipids from coarse-grained molecular dynamics simulations of tBamA in bilayers of different thicknesses. Data are shown for bulk lipids (those greater than 30 Å from tBamA), and those within 12 Å of K808, at the β1-β16 seam of tBamA, and L613 on the opposite side of the barrel (**Fig. S8e**). Data shown are the mean ± standard deviation from 5 independent simulations. The duration of each simulation was 3 μs and data analysis was performed on the final 2.5 μs.

| **Lipid type** | **Bond** | **Lipid region** | **<P_2_> order parameter** |
| --- | --- | --- | --- |
| *di*C8:0-10:0 | G-1 | Bulk | 0.48 ± 0.0002 |
|  | G-1 | L79 | 0.42 ± 0.005 |
|  | G-1 | L164 | 0.44 ± 0.01 |
|  | 1-2 | Bulk | 0.35 ± 0.0005 |
|  | 1-2 | L79 | 0.30 ± 0.005 |
|  | 1-2 | L164 | 0.30 ± 0.01 |
| *di*C12:0-14:0 | G-1 | Bulk | 0.51 ± 0.0003 |
|  | G-1 | L79 | 0.46 ± 0.007 |
|  | G-1 | L164 | 0.46 ± 0.01 |
|  | 1-2 | Bulk | 0.40 ± 0.0003 |
|  | 1-2 | L79 | 0.33 ± 0.005 |
|  | 1-2 | L164 | 0.33 ± 0.007 |
|  | 2-3 | Bulk | 0.36 ± 0.0008 |
|  | 2-3 | L79 | 0.29 ± 0.006 |
|  | 2-3 | L164 | 0.29 ± 0.008 |
| *di*C16:0-18:0 | G-1 | Bulk | 0.51 ± 0.0005 |
|  | G-1 | L79 | 0.45 ± 0.01 |
|  | G-1 | L164 | 0.46 ± 0.03 |
|  | 1-2 | Bulk | 0.46 ± 0.0009 |
|  | 1-2 | L79 | 0.38 ± 0.01 |
|  | 1-2 | L164 | 0.37 ± 0.02 |
|  | 2-3 | Bulk | 0.38 ± 0.0008 |
|  | 2-3 | L79 | 0.30 ± 0.009 |
|  | 2-3 | L164 | 0.29 ± 0.02 |
|  | 3-4 | Bulk | 0.32 ± 0.0007 |
|  | 3-4 | L79 | 0.23 ± 0.008 |
|  | 3-4 | L164 | 0.22 ± 0.02 |

**Table S8.** <P_2_> bond order parameters of lipids from coarse-grained molecular dynamics simulations of tOmpA in bilayers of different thicknesses. Data are shown for bulk lipids (those greater than 30 Å from tOmpA), and those within 12 Å of L79, at the β1-β8 seam of tOmpA, and L164 on the opposite side of the barrel (**Fig. S8d**). Order parameters values are shown for the bonds between glycerol particles and first lipid acyl chain particles (G-1), and between consecutive lipid acyl chain particles (1-2, 2-3, and 3-4). Data shown are the mean ± standard deviation from 5 independent simulations. The duration of each simulation was 3 μs and data analysis was performed on the final 2.5 μs.

| **Lipid type** | **Lipid region** | **Membrane thickness (Å)** |
| --- | --- | --- |
| *di*C8:0-10:0 | Bulk | 27.0 ± 0.1 |
|  | L79 | 24.7 ± 0.3 |
|  | L164 | 24.8 ± 0.3 |
| *di*C12:0-14:0 | Bulk | 33.9 ± 0.008 |
|  | L79 | 30.0 ± 0.3 |
|  | L164 | 29.8 ± 0.2 |
| *di*C16:0-18:0 | Bulk | 40.6 ± 0.03 |
|  | L79 | 35.0 ± 0.5 |
|  | L164 | 34.5 ± 0.7 |

**Table S9.** Membrane thickness of lipids from coarse-grained molecular dynamics simulations of tOmpA in bilayers of different thicknesses. Data are shown for bulk lipids (those greater than 30 Å from tOmpA), and those within 12 Å of L164, at the β1-β8 seam of tOmpA, and L79 on the opposite side of the barrel (**Fig. S8d**). Data shown are the mean ± standard deviation from 5 independent simulations. The duration of each simulation was 3 μs and data analysis was performed on the final 2.5 μs.

| **BamA construct** | **Additions** | **t_50_ value (min)** |
| --- | --- | --- |
| Wild-type | None | 22.3 ± 1.5 |
|  | CuSO_4_ | 15.7 ± 1.1 |
|  | TCEP | 7.9 ± 0.7 |
| Cys-free | None | 18.8 ± 0.6 |
|  | CuSO_4_ | 18.6 ± 2.0 |
|  | TCEP | 12.3 ± 1.0 |
| X-link | None | 17.3 ± 1.7 |
|  | CuSO_4_ | 16.8 ± 1.3 |
|  | TCEP | 8.5 ± 0.5 |

**Table S10:** Measured t_50_ values for tOmpA folding into DMPC liposomes in the presence of BamA, BamA^Cys-free^ or BamA^X-link^. Samples contained 0.4 μM tOmpA, 1.28 mM DMPC, 0.24 M urea, 50 mM glycine-NaOH, pH 9.5, at 30 ºC. A two-fold molar excess (0.8 μM) of wild-type BamA, BamA^Cys-free^ or BamA^X-link^ was used. In samples containing CuSO_4_ or TCEP, concentrations of 1 mM and 25 mM were used, respectively. Data are shown as the mean ± the standard error of the mean (s.e.m.) of the t_50_ values obtained from a minimum of three separate folding experiments, each using independently prepared batches of liposomes.

**Supplementary References**

[1] N.K. Burgess, T.P. Dao, A.M. Stanley, K.G. Fleming, β-barrel proteins that reside in the Escherichia coli outer membrane in vivo demonstrate varied folding behavior in vitro, J. Biol. Chem. 283 (2008) 26748–26758.

[2] B.M. Burmann, C. Wang, S. Hiller, Conformation and dynamics of the periplasmic membrane-protein–chaperone complexes OmpX–Skp and tOmpA–Skp, Nat Struct Mol Biol. 20 (2013) 1265–1272.

[3] Swiss Institute of Bioinformatics, The ExPASy ProtParam webserver, http://web.expasy.org/protparam/.

[4] P.G. Blommel, B.G. Fox, A combined approach to improving large-scale production of tobacco etch virus protease, Protein Expression and Purification. 55 (2007) 53–68.

[5] E.J. Danoff, K.G. Fleming, Membrane defects accelerate outer membrane β-barrel protein folding, Biochemistry. 54 (2015) 97–99.

[6] A. Pautsch, G.E. Schulz, High-resolution structure of the OmpA membrane domain, J. Mol. Biol. 298 (2000) 273–282.

[7] A. Fiser, R.K. Do, A. Sali, Modeling of loops in protein structures, Protein Sci. 9 (2000) 1753–1773.
